# Implementation Strategies for Promoting Physical Activity in Under-Resourced South African Schools: A Scoping Review

**DOI:** 10.3390/children13101289

**Published:** 2026-09-22

**Authors:** Livhuwani Phylist Ramashiya, Musa Lewis Mathunjwa, Lebogang Faith Thaga, Gudani Goodman Mukoma

**Affiliations:** 1 Human Movement Science Department, Faculty of Science, Agriculture & Engineering, University of Zululand, KwaDlangezwa 3886, South Africa; 2 Department of Biokinetics, Recreation and Sports Science, Faculty of Health Sciences, University of Venda, Thohoyandou 0950, South Africa; lebogang.thaga@univen.ac.za

**Keywords:** curriculum-integrated physical activity, implementation barriers, physical education, school-based physical activity, under-resourced schools, South Africa

## Abstract

**Highlights:**

**What are the main findings?**

- Curriculum-integrated physical activity approaches, including Physical Education, Life Orientation activities, classroom activity breaks, and multicomponent programmes, were found to be commonly reported in under-resourced South African schools.
- Teacher training, learner engagement, stakeholder support, and low-cost resources were frequently reported facilitators, while resource and implementation-capacity constraints were found to be common barriers.

**What are the implications of the main findings?**

- Physical activity promotion in under-resourced schools may be strengthened through curriculum integration, educator support, stakeholder engagement, and contextually appropriate low-cost implementation strategies.
- Future research should evaluate implementation fidelity, sustainability, scalability, and interventions targeting secondary-school learners.

**Abstract:**

**Background/Objectives:** Learners in under-resourced South African schools face substantial barriers to physical activity (PA). Schools provide a feasible platform for PA promotion in disadvantaged communities where alternative recreational opportunities are scarce. This scoping review synthesised implementation strategies, barriers, facilitators, and evidence gaps regarding school-based PA promotion in under-resourced South African schools. **Methods:** Guided by the Joanna Briggs Institute methodology, this review is reported following PRISMA-ScR and PRISMA-S guidelines. PubMed, Scopus, Web of Science, and EBSCOhost (SPORTDiscus, ERIC, CINAHL, and Academic Search Complete) were searched as electronic databases, while Google Scholar (via Publish or Perish) was searched separately as a supplementary source for eligible peer-reviewed studies and the formal institutional grey literature published between January 2010 and June 2026. Eligible studies examined structured PA interventions in government-funded primary and secondary schools in under-resourced settings. Data were extracted using a standardised form, synthesised descriptively, and appraised using the appropriate Joanna Briggs Institute critical appraisal checklists. **Results:** Twenty-four studies met the inclusion criteria. Most interventions were curriculum-integrated and delivered during school hours through Physical Education, Life Orientation, classroom activity breaks, or multicomponent programmes. Common barriers included inadequate infrastructure, limited teacher capacity, competing academic demands, constrained instructional time, and challenges in implementation fidelity. Key facilitators included curriculum alignment, teacher training and support, learner engagement, provision of low-cost resources, and culturally appropriate programme delivery. **Conclusions:** This review mapped the implementation strategies for school-based physical activity in under-resourced South African schools. Curriculum integration, educator support, stakeholder engagement, and low-cost, contextually appropriate approaches were reported as facilitators of programme implementation, whereas resource constraints and limited implementation capacity remained persistent barriers. Given the heterogeneity of the evidence, these findings should be interpreted as reported influences of implementation rather than as established determinants of programme success. Future research should strengthen the evidence base by evaluating interventions in secondary schools and examining implementation fidelity and long-term sustainability.

## 1. Introduction

Regular PA is vital for children’s growth, supporting physical health, psychosocial development and academic performance [1,2,3]. Despite this, learners in under-resourced rural schools often face barriers to PA, including inadequate infrastructure, curriculum constraints, limited teacher training, and unsafe environments [4,5,6]. These challenges are especially pronounced in rural communities, where issues of safety, poverty, and limited recreational spaces further restrict participation [7]. Teacher capacity also remains a challenge, as many educators responsible for Life Orientation (LO) lack the training and confidence to deliver effective physical education lessons [5,8,9].

Whole-school and multicomponent approaches have been shown internationally to enhance PA participation, providing a framework for supporting disadvantaged schools in South Africa [2,3,6]. Additionally, physical literacy frameworks offer a global foundation for improving children’s long-term engagement in movement [10,11]. A recent scoping review on school-based physical literacy promotion highlights that most interventions are delivered through physical education and curriculum-integrated activities, but the evidence remains heterogeneous and underdeveloped in low-resource school contexts [10].

Several school-based interventions, including KaziKidz and DASH, have been implemented in South African schools, yet the evidence is often fragmented, short-term, or not tailored to rural and disadvantaged contexts [12,13]. An umbrella review of school-based physical activity interventions found that curriculum-embedded and multicomponent programmes show the most consistent short-term benefits, although long-term sustainability and contextual adaptation in low-resource settings remain limited [14].

Although numerous school-based physical activity interventions have demonstrated effectiveness under controlled research conditions, successful implementation within routine educational practice requires consideration of contextual, organisational, and behavioural influences [15,16]. Implementation science provides theoretical frameworks that facilitate understanding of why interventions succeed or fail when translated into real-world settings [17]. The Consolidated Framework for Implementation Research (CFIR) identifies five interacting domains that influence implementation: intervention characteristics, the outer setting, the inner setting, characteristics of individuals, and implementation processes [18,19]. Although none of the included studies explicitly applied CFIR, it was used as a sensitising framework to support the interpretation of reported implementation barriers and facilitators. It was not used as a formal coding framework or to classify study findings, and the conceptual synthesis was derived from the factors reported in the included studies.

In South Africa, these challenges are particularly evident in under-resourced schools, where overcrowding, inadequate facilities, limited teacher preparation, and competing academic priorities may restrict opportunities for meaningful physical activity participation [20,21,22].

Previous reviews have examined school-based physical activity from different perspectives, including intervention effectiveness and physical activity and fitness outcomes [6], as well as school-based physical literacy promotion [10]. International evidence has also synthesised factors influencing the adoption, implementation, and sustainability of school-based physical activity interventions in real-world settings [23]. Within sub-Saharan Africa, a scoping review has mapped evidence relating to physical activity recommendations and promotion, including studies conducted in South Africa [24]. A South Africa-specific synthesis is warranted because the implementation of school-based physical activity promotion occurs within a distinct national education, socioeconomic, and resource context, with substantial inequalities in school infrastructure and capacity. However, there remains limited synthesis focused specifically on how physical activity promotion strategies are implemented, adapted, and sustained within under-resourced South African primary and secondary schools.

Consequently, important questions remain regarding how schools with limited infrastructure, constrained human resources, and competing educational priorities adopt, deliver, and sustain physical activity interventions. Addressing these knowledge gaps is important for informing contextually appropriate and potentially scalable implementation strategies to strengthen physical activity promotion in under-resourced South African schools.

To address this gap, a scoping review was conducted to systematically map existing evidence and identify implementation-related factors relevant to disadvantaged school settings. Specifically, this review aimed to: (1) map physical activity promotion strategies implemented among learners in under-resourced primary and secondary schools in South Africa; and (2) identify the barriers, facilitators, and evidence gaps influencing their implementation.

## 2. Materials and Methods

### 2.1. Protocol and Reporting Framework

This scoping review was conducted in accordance with the Joanna Briggs Institute (JBI) methodology for scoping reviews and reported following the PRISMA-ScR and PRISMA-S guidelines [25,26,27]. Although the review protocol was not prospectively registered, the review methodology, eligibility criteria, search strategy, screening procedures, and data-charting framework were established a priori before commencement of study selection. While prospective registration enhances methodological transparency and minimises the risk of post hoc methodological modifications, protocol registration is not considered mandatory for scoping reviews conducted in accordance with the Joanna Briggs Institute guidance. The absence of protocol registration is nevertheless acknowledged as a limitation when interpreting the methodological transparency of this review.

### 2.2. Eligibility Criteria

Eligibility criteria were defined using the PCC framework:

Population (P): Learners aged 5–18 years (or those actively enrolled in the primary or secondary school phases) attending government-funded, in-person schools in South Africa.

Concept (C): Structured school-based PA promotion strategies or interventions (e.g., curriculum-based PE, extracurricular sports, or whole-school initiatives). This review focuses specifically on structured interventions to enable consistent mapping of intervention components and their delivery strategies.

Context (C): Under-resourced or disadvantaged school settings, including rural and low-income communities in South Africa. For this review, a school was considered under-resourced or disadvantaged only when the study reported sufficient contextual evidence of socioeconomic disadvantage and/or resource limitations relevant to physical activity provision. Indicators included explicit descriptions of under-resourced, low-income, low-resource, resource-constrained, or resource-poor settings; disadvantaged or underserved communities; poverty or impoverishment; low socioeconomic status; documented limitations in facilities, equipment, or infrastructure; school-quintile classification; and no-fee school status. Rural, township, urban, or peri-urban location alone was not considered sufficient evidence of an under-resourced context, and school-quintile classification or no-fee status was not treated as determinative in isolation. Eligibility was based on the overall contextual evidence reported for the participating schools. For studies involving mixed-resource school populations, inclusion required sufficient evidence that the schools or participants were representative of the defined under-resourced context.

#### Inclusion and Exclusion Criteria

Inclusion criteria comprised empirical studies (including final ahead-of-print or early online publications with a registered DOI) and eligible grey literature, published in English between January 2010 and June 2026, and conducted strictly within South Africa; in-person, government-funded primary and secondary schools serving under-resourced or disadvantaged communities and targeting learners aged 5–18 years (or those actively enrolled in the two phases); and structured school-based PA interventions or promotion strategies, including Physical Education programmes, sport participation programmes, physical literacy interventions, motor competence interventions, fundamental movement skills (FMSs) programmes, and school-based implementation strategies, programmes, initiatives, approaches, or interventions reporting outcomes related to PA levels, health markers, or implementation metrics.

Exclusion criteria included studies focusing on adults, university or college students, or preschool children; studies not conducted in school settings; cyber or home-schooling contexts; studies reporting only physical activity prevalence or levels, barriers, facilitators, perceptions, or correlates without an intervention or implementation component; treatment, rehabilitation, or clinical exercise programmes; studies conducted outside South Africa or in private, well-resourced, high-income, or non-school settings; reviews (including systematic reviews, scoping reviews, narrative reviews, literature reviews, umbrella reviews, and meta-analyses); editorials, commentaries, opinion papers, news articles, blogs, press releases, fact sheets, and conference abstracts or conference posters without sufficient methodological information; non-English publications; and publications published before 2010.

The restriction to English-language publications was applied to facilitate accurate interpretation of findings and maintain consistency during data extraction.

### 2.3. Information Sources and Search Strategy

A comprehensive search was conducted across four electronic databases: PubMed (via NCBI), Scopus (Elsevier), Web of Science (Clarivate Analytics), and EBSCOhost (SPORTDiscus, ERIC, CINAHL, and Academic Search Complete). Google Scholar (via Publish or Perish) was searched separately as a supplementary source. All databases were searched on 16 June 2026, covering publications from January 2010 to June 2026. The search strategy was developed in accordance with the PRISMA-S [28] reporting guideline and combined controlled vocabulary (e.g., MeSH terms in PubMed) with free-text keywords related to the following concepts: physical activity, promotion/intervention, learners/children/adolescents, school settings, and under-resourced contexts. South Africa was not included as a geographical search term because the search strategy was designed to maximise sensitivity and minimise the risk of missing relevant studies that did not explicitly identify South Africa in the title, abstract, or indexed fields. The geographical eligibility criterion was therefore applied during title and abstract screening and confirmed during full-text screening, with eligible studies required to have been conducted in South Africa.

Search strategies were tailored for each database, employing database-specific syntax, truncation, and field tags to optimise both sensitivity and retrieval effectiveness.

Detailed search strategies for all databases are provided in Supplementary Table S3 to ensure transparency and reproducibility. Search parameters were set to include only English-language studies published from 2010 to 2026. The language criterion was established to facilitate accurate interpretation and aligns with the primary medium of instruction and research publication in South African school contexts. Publications from 2010 were selected to capture contemporary evidence on school-based physical activity promotion and implementation strategies in South African school settings, while ensuring sufficient coverage of developments over time.

For Google Scholar, searches were conducted using Publish or Perish (version 8) to enable structured retrieval and effective result management, with the first 200 results sorted by relevance screened [29,30]. While Google Scholar was utilised to capture official institutional reports and policy documents (grey literature) relevant to South African schooling, the informal grey literature (such as opinion pieces or commentary) was excluded. The review included peer-reviewed evidence and eligible formal institutional publications to ensure methodological transparency and verifiable reporting. Finally, reference lists of the selected studies were screened to identify additional relevant literature.

Example of search strategy: Scopus

TITLE-ABS-KEY

(implementation OR intervention* OR strateg* OR promotion OR “health promotion” OR program* OR programme* OR initiative* OR “implementation science”)

AND

(“physical activity” OR exercise OR sport* OR “sport participation” OR “physical education” OR PE OR “physical literacy” OR “motor competence” OR “motor skill*” OR “movement skill*” OR “fundamental movement skill*” OR FMS OR “active classroom*” OR “active school*” OR “Life Orientation” OR LO OR “school health” OR “school wellness”)

AND

(learner* OR child* OR children OR student* OR adolescent* OR youth)

AND

(“under-resourced” OR “low-income” OR “low-resource” OR rural OR disadvantaged OR underserved OR township* OR “resource-constrained” OR “resource poor” OR “low socioeconomic” OR “low SES” OR poverty OR impoverished OR quintile* OR “no-fee school*”)

AND

(school*)

The standalone search terms “PE” (Physical Education) and “LO” (Life Orientation) were used because they are commonly used abbreviations within the South African education system and are frequently used to describe curriculum-based physical activity programmes in schools. These terms were included to maximise the sensitivity of the search strategy and to minimise the risk of missing relevant studies that may have used the abbreviations rather than the full terms.

### 2.4. Selection of Sources of Evidence

All retrieved records were imported into Covidence (Veritas Health Innovation, Melbourne, Australia) for duplicate removal and screening [31]. Screening occurred in two sequential stages: (1) title and abstract screening and (2) full-text review.

To ensure consistency, two reviewers independently screened all records against the predefined eligibility criteria. Prior to formal screening, calibration exercises were conducted to align the interpretation of the inclusion criteria. Disagreements at either stage were resolved through discussion and consensus between the two reviewers. Screening agreement was therefore assessed through calibration and consensus-based resolution of disagreements; no formal inter-rater agreement statistic was calculated. This process was designed to maximise transparency and minimise selection bias.

### 2.5. Data Charting Process

A standardised data charting form was developed a priori and piloted on five studies to evaluate its completeness and ease of use. The form was refined iteratively throughout the data charting process to capture unexpected nuances in the data and minimise extraction bias. Two reviewers independently charted data from the included studies. The charted data comprised study characteristics (e.g., author, year, location), intervention components, target outcomes, and specific contextual factors relevant to school implementation. Discrepancies between reviewers were resolved through consensus or, where necessary, consultation with a third reviewer.

### 2.6. Data Items

The following variables were charted from each included study:

- Study characteristics: Author(s), year of publication, country (South Africa), study design, and sample size.
- Population details: Participant age range, school grade levels, and school type (e.g., primary, secondary, or combined).
- Concept (intervention/strategy): Type of PA promotion initiative (e.g., curriculum-aligned PE, after-school programs, active classroom breaks, or whole-school PA approaches).
- Contextual factors: Description of the under-resourced or rural setting, socioeconomic indicators of the community, and infrastructural factors (e.g., availability of sports fields, equipment, or facilities).
- Outcomes: Measures of learner PA participation, physical fitness levels, health indicators, and psychosocial well-being.
- Barriers and facilitators: Structural, social, institutional, or systemic factors reported as influencing the implementation or sustainability of the intervention.

### 2.7. Critical Appraisal of Individual Sources of Evidence

A methodological quality appraisal was conducted to provide context for interpreting the evidence in this scoping review and to evaluate the strengths and limitations of the included studies. Each study was appraised using the appropriate JBI Critical Appraisal Checklist based on its design [25,32].

The first author conducted the critical appraisal using the relevant JBI checklist. The completed appraisals were subsequently reviewed by the second reviewer and the research supervisors for consistency and accuracy; therefore, a critical appraisal was not conducted independently in duplicate. The appraisal results helped inform the interpretation of findings, but no studies were excluded due to quality issues. The purpose of critical appraisal was to inform readers about the methodological strengths and limitations, not to determine study eligibility. In line with Joanna Briggs Institute guidelines for scoping reviews, no studies were excluded on quality grounds; instead, the appraisals highlighted the overall strength of the evidence and areas needing future high-quality implementation research.

### 2.8. Synthesis of Results

The charted data were synthesised using descriptive and narrative approaches. Included studies were categorised as either reporting structured school-based physical activity interventions or examining implementation-related factors.

The synthesised findings were mapped across four core evidence domains:

1. Typologies of physical activity interventions and strategies;
2. Barriers to school-based implementation;
3. Reported facilitators of implementation;
4. Identified evidence gaps in the current literature.

The results are presented using tabular summaries detailing the characteristics and findings of the included sources, supplemented by a structured narrative synthesis to maximise clarity and interpretability.

CFIR was identified a priori as a sensitising framework to inform the interpretation of implementation-related barriers and facilitators reported in the included studies. It was not used as a formal coding framework or to classify study findings; rather, it provided a conceptual lens for interpreting the implementation-related evidence.

## 3. Results

### 3.1. Selection of Sources of Evidence

The database searches identified 15,245 records across PubMed (*n* = 2826), Scopus (*n* = 7942), Web of Science (*n* = 278), and EBSCOhost (*n* = 4199). An additional 1213 records were identified through Google Scholar as supplementary sources.

The substantially higher number of records retrieved from Scopus compared with Web of Science reflects differences in database coverage, indexed source titles, searchable fields, and search syntax. Scopus searches were conducted using the TITLE-ABS-KEY field, which includes a broader range of indexed journals, whereas Web of Science uses different indexing structures and field-tag specifications. Therefore, retrieval differences should not be interpreted as differences in the underlying evidence base. After removing 1121 duplicates, 15,339 records were screened at the title and abstract level. Of these, 174 reports were sought for retrieval, two reports could not be retrieved, and 172 reports were assessed for eligibility. Twenty-four studies met all inclusion criteria. Reasons for full-text exclusion included ineligible population, context, intervention or concept, country, publication type, study design, insufficient methodological and intervention details, unclear context/settings, mixed population, and mixed context.

When the study context was unclear during the eligibility assessment, the corresponding authors were contacted via email for clarification. Five authors were contacted; four responded, and one did not. Information obtained through correspondence with the author confirmed the eligibility of one study for inclusion in the review. The remaining studies were excluded because they did not meet the eligibility criteria for the review.

The complete study selection process is mapped out in the PRISMA-ScR flow diagram (Figure 1).

### 3.2. Characteristics of Sources of Evidence

The twenty-four included studies employed a variety of methodological designs, including randomised controlled trials (*n* = 12), quasi-experimental studies (*n* = 11), and prospective cohort studies (*n* = 1). These studies are presented in Supplementary Table S1. Reported sample sizes ranged from 41 to 1304 participants. These values reflect the sample sizes reported in the individual studies; because reporting conventions differed across studies, sample sizes were not standardised to baseline enrolment or analytic samples. The interventions primarily targeted primary school learners aged approximately 5–17 years, spanning Grades R to 7, although some studies included adolescents.

Geographically, the evidence was distributed across six South African provinces, with the Eastern Cape contributing the largest number of studies (*n* = 13), followed by the Western Cape (*n* = 5), North West Province (*n* = 3), Gauteng (*n* = 1), KwaZulu-Natal (*n* = 1), and the Free State (*n* = 1). No eligible studies were identified from the Northern Cape, Mpumalanga, and Limpopo. Importantly, although 24 studies were included, several studies reported findings from the same intervention programmes and school populations. Consequently, the number of unique implementation programmes was smaller than the number of included publications, although a definitive number of unique school sites could not be established because school identifiers were not consistently reported across publications. Supplementary Table S4 clearly indicates the Programme Clusters and Sample Overlap.

Methodologically, most interventions were implemented in low-income public schools located in rural, township, peri-urban, or other resource-constrained settings. The baseline characteristics of the included studies are summarised in Supplementary Table S1.

### 3.3. Critical Appraisal Within Sources of Evidence

Critical appraisal was undertaken using the appropriate Joanna Briggs Institute (JBI) critical appraisal checklists according to study design, including randomised controlled trials, quasi-experimental studies, and prospective cohort studies (Supplementary Table S2A–C).

Overall, the included studies demonstrated varying methodological strengths and limitations. Common strengths included clearly defined study populations, standardised outcome measures, and appropriate assessment procedures [33,34,35,36,37,38,39].

Among the intervention studies, the most frequently identified limitations were non-randomised or quasi-experimental designs, convenience or purposive sampling, baseline differences between intervention and control groups, and incomplete follow-up [40,41,42,43,44,45,46,47,48,49,50,51]. These limitations may have reduced group comparability and the ability to attribute observed changes solely to the interventions.

No studies were excluded on the basis of methodological quality, as the purpose of the critical appraisal was to describe the overall strengths and limitations of the available evidence and to inform interpretation of the review findings.

### 3.4. Results of Individual Sources of Evidence

#### 3.4.1. Physical Activity Promotion Strategies

The 24 included studies evaluated a range of school-based physical activity (PA) promotion strategies, with considerable variation in intervention content, delivery, duration, and level of implementation support. Because several studies originated from the same programmes or school samples, the findings below should be interpreted as evidence from related studies rather than as 24 independent intervention populations.

Curriculum-integrated approaches were the most frequently reported strategy. These included structured Physical Education (PE), Moving-to-Music sessions, classroom activity breaks, and curriculum-linked movement activities. The KaziKidz/KaziAfya publications incorporated PE, Moving-to-Music, classroom activity breaks, teacher training, and curriculum-aligned resources, with delivery supported by PE coaches or other trained personnel [33,34,35,36,39]. Similarly, Van der Merwe et al. [52] used the KaziKidz toolkit to deliver curriculum-aligned perceptual-motor activities during allocated PE periods.

Enhanced PE approaches were also reported. Tian, du Toit and Toriola [48,49] evaluated the same enhanced PE programme in two low-socioeconomic-status primary schools in Potchefstroom. The programme incorporated physical fitness activities, indigenous games, improvised equipment, homework activities, a reward system, and monitoring of activity intensity within the CAPS curriculum. The 2014 study focused primarily on physical and motor fitness outcomes, whereas the 2017 study examined physical activity and sedentary behaviour outcomes.

A second group of interventions focused specifically on fundamental movement skills (FMSs) and motor competence. Three studies by Idamokoro et al. [44,45,46] evaluated SPARK-derived FMS programmes involving locomotor, ball, balance, and aerobic activities in rural Eastern Cape schools. The studies reported improvements in selected FMSs and, in the 2023 study, body-composition measures, while the intervention did not produce a corresponding improvement in moderate-to-vigorous physical activity. The six-month follow-up provided evidence of sustained improvements in motor competence. Africa and Van Deventer [40] used a structured motor-skills programme focusing on balance and bilateral coordination, while Africa et al. [41] evaluated a short gross-motor intervention incorporating locomotor, object-control, and stability skills. The inclusion of Grade R learners from Africa, as noted by van Stryp and Vermaak [41], was considered appropriate because the study was conducted in a Quintile 2 government primary school, and the Grade R learners were enrolled in a formal primary school setting rather than a preschool or early childhood development centre. Therefore, they did not meet the preschool exclusion criterion for the review.

Multicomponent strategies combined PA with other health-promotion activities or environmental changes. Nqwenosi et al. [13,37,38,53], together with [54], described interventions combining PE, Moving-to-Music, classroom activity breaks, and modifications to the school environment, such as painted playground games, activity stations, or play structures. Nqweniso et al. [37] additionally incorporated health education, deworming, and nutritional supplementation. These publications were conducted in related disadvantaged school settings and therefore represent a programme cluster rather than wholly independent evidence.

Other approaches used sport, after-school activity, or broader school health-promotion models. Fuller [55] implemented a football-based programme combining football skills with health education during school time. Zimu et al. [51] combined a school-based social-marketing campaign with an after-school programme involving cardio dance and muscular-endurance circuit training. Pienaar et al. [47] used aerobic exercise, ball games, and strength and flexibility activities for adolescents. Williams [50] implemented a broader school health-promotion programme incorporating physical fitness, healthy eating, and psychosocial activities, although PA or fitness outcomes were not reported. Williams [50] met the review’s eligibility criteria for grey literature because it was an academic thesis reporting a school-based intervention in an under-resourced school setting and provided sufficient methodological information for data extraction. No other eligible theses meeting the review’s inclusion criteria were identified during the screening process.

Ferguson [42] used a whole-school health-promotion approach incorporating PE, playground activities, equipment, environmental adaptations, and educator collaboration. Although the sample included learners with and without developmental coordination disorder, the study was retained because the intervention was implemented as a school-based physical activity programme within a low-income primary school rather than as a clinical rehabilitation or treatment intervention.

Fisher and Africa [43] integrated PA and perceptual-motor activities into mathematics lessons.

Delivery arrangements varied considerably. Most interventions were implemented during normal school hours and relied on classroom teachers, PE specialists, university students, coaches, or research personnel. Resources included structured toolkits and lesson plans, sports equipment, improvised materials, playground markings, and environmental adaptations. After-school delivery was reported less frequently, with Zimu et al. [51] providing a clear example of an adolescent-focused after-school model.

Overall, the included evidence demonstrates substantial heterogeneity in how PA was promoted, ranging from curriculum-integrated PE and classroom activities to FMS programmes, sport-based interventions, environmental modifications, and multicomponent health-promotion approaches. The available studies, therefore, do not support identifying a single implementation strategy as superior across under-resourced South African school contexts. Rather, the evidence indicates variation in intervention intensity, duration, delivery personnel, contextual adaptation, and implementation support.

#### 3.4.2. Barriers to Implementation

The included studies reported several contextual, resource-related, and organisational barriers to the implementation of school-based physical activity programmes:

- Resource and infrastructure constraints: Limited access to appropriate sports facilities, inadequate playground or activity spaces, shortages of physical education equipment, and the need to use improvised or low-cost resources presented challenges to programme delivery in several under-resourced school settings [38,39,40,42,44,48,49].
- Human resource and capacity limitations: Limited availability of appropriately trained Physical Education personnel, teacher capacity, competing teaching responsibilities, and insufficient time for programme delivery were reported as challenges. Several interventions, therefore, relied on external support from PE specialists, coaches, university students, researchers, or other trained personnel to facilitate implementation [13,34,38,39,42,47,48,49,53].
- Implementation disruptions: Learner absenteeism, difficulties maintaining attendance, adverse weather conditions, community protests, changes to the school calendar, early school closure, and disruption associated with the COVID-19 pandemic affected the continuity or delivery of some interventions [33,36,39,43,47,52].
- Limited stakeholder engagement and competing school priorities: Limited involvement or support from teachers, parents, or other school stakeholders, together with competing educational priorities, was reported as a challenge in some interventions. These factors could constrain the delivery of planned activities and the extent to which interventions were integrated into routine school practices [38,39,42,43,50].

#### 3.4.3. Facilitators of Implementation

Conversely, several implementation approaches were reported as supporting delivery acceptability within under-resourced schools:

- Curriculum integration: Aligning physical activity interventions with the existing school curriculum, including Physical Education, Life Orientation, and classroom lessons, facilitated integration into the school day. Curriculum-aligned approaches enabled physical activity to be incorporated into existing teaching structures rather than requiring completely separate programme periods [33,34,35,36,38,39,40,43,48,49,52].
- Training and implementation support: Teacher training, structured lesson plans, intervention manuals, coaching, and support from researchers, PE specialists, university students, and other trained personnel facilitated programme delivery. These forms of support were particularly relevant where schools had limited access to specialised Physical Education personnel [13,34,38,39,44,45,46,48,49,52,53].
- Contextually appropriate and low-cost approaches: The use of affordable or improvised equipment, activities adapted to available school facilities, culturally familiar activities, and flexible delivery approaches supported implementation in settings with limited resources. Examples included indigenous games, dance and music-based activities, playground adaptations, and the use of available or low-cost materials [38,39,40,44,45,48,49,51,55].
- School, stakeholder, and learner engagement: Engagement of teachers, school leadership, parents, community members, and learners supported participation and programme acceptability in some settings. Learner-centred activities, including games, music, dance, and motivational strategies, were also used to encourage participation and engagement [38,39,40,44,45,50,51,55].

#### 3.4.4. Identified Evidence Gaps

Several clear boundaries persist in the current South African literature landscape:

- Population coverage: Most studies focused on primary school learners, with comparatively fewer studies involving secondary school learners or adolescents. Only Pienaar et al. [47] and Zimu et al. [51] specifically focused on adolescent or secondary-school populations. This limits the available evidence on the implementation of physical activity promotion strategies among older learners.
- Geographical distribution: The included studies were conducted across several South African provinces, but evidence was concentrated in the Eastern Cape, with additional studies from the Western Cape, Northwest, Gauteng, KwaZulu-Natal, and Free State. Evidence from other provinces and from more remote rural settings remained limited.
- Implementation fidelity and sustainability: Few studies provided a detailed assessment of implementation fidelity, maintenance, scalability, or longer-term delivery beyond the intervention period. Although some studies reported attendance, intervention adherence, implementation support, or follow-up outcomes, these measures were not consistently applied across the evidence base [33,36,39,44,45,47,51].
- Implementation-process evidence: Most included studies primarily evaluated intervention or participant outcomes, while fewer provided a detailed examination of implementation processes, stakeholder experiences, organisational factors, or contextual influences on programme delivery. Consequently, evidence remains limited regarding how interventions are adopted, implemented, maintained, and adapted within routine school practice [36,37,39,51,53,54].
- Evidence of independent programme effects: Several studies described related intervention programmes, school settings, or samples, including the KaziKidz/KaziAfya programmes [33,34,35,36,39], the multicomponent school-based PA/DASH interventions [13,37,38,53,54], the SPARK-adapted FMS programme [44,45] and the enhanced PE intervention [48,49]. Therefore, the 24 studies should not be interpreted as representing 24 completely independent intervention populations. The extent of confirmed sample overlap varied between programme clusters, highlighting the need for future research to clearly report programme identifiers, school sites, participant cohorts, and linked studies.

#### 3.4.5. Thematic Synthesis of Implementation Findings

Table 1 presents a synthesis of the principal implementation themes identified across the 24 included studies. Physical activity programmes were most commonly delivered through curriculum-integrated approaches and structured motor skill activities incorporated into the school day. Curriculum alignment, contextual adaptation, teacher training and implementation support, and stakeholder engagement were reported as important implementation considerations. However, inadequate infrastructure, limited equipment, competing teaching responsibilities and insufficient teacher capacity were consistently identified as barriers to programme delivery.

The evidence also revealed comparatively fewer interventions conducted in secondary school settings and limited evaluation of implementation fidelity, long-term sustainability, maintenance, and scalability. Several studies were also linked through common programmes, schools, or participant cohorts, indicating that the 24 studies did not necessarily represent 24 independent intervention populations.

### 3.5. Summary of Implementation Fidelity and Sustainability

Although implementation fidelity was reported in several included studies, relatively few formally assessed fidelity using predefined indicators. Studies mainly reported teacher training, implementation manuals, curriculum-aligned lesson plans, external support, attendance, or adherence [33,36,38,39,44,45,46].

Implementation was also affected by contextual factors, including competing curriculum demands, limited instructional time, inadequate infrastructure, school-calendar disruptions, and COVID-19. Evidence on long-term sustainability, maintenance, and scalability remained limited across the included studies.

### 3.6. Overall Synthesis of Implementation Findings

Across the included studies, five recurring implementation characteristics were identified:

- Curriculum integration;
- Educator training and ongoing implementation support;
- Provision of low-cost physical activity resources;
- Leadership and stakeholder engagement;
- Contextual adaptation to local school environments.

Conversely, limited educator preparation, inadequate infrastructure, competing school priorities, resource constraints, and insufficient implementation monitoring were reported as barriers to implementation. Overall, the reported implementation experiences reflected both intervention characteristics and the organisational and contextual conditions of under-resourced school settings. Because several publications were linked through common programmes, schools, or participant cohorts, these recurring characteristics should be interpreted as reported implementation considerations rather than independent determinants of programme success.

## 4. Discussion

This scoping review synthesised reported implementation strategies, barriers, facilitators, and evidence gaps relating to school-based physical activity programmes in under-resourced South African schools. Across the 24 included publications, implementation experiences varied with intervention requirements and the organisational and contextual conditions under which programmes were delivered. The findings suggest that implementation may be more feasible when physical activity can be incorporated into existing school routines and supported by appropriate organisational resources. However, given the heterogeneity of the evidence and limited assessment of implementation fidelity and sustainability, these findings should be interpreted as reported implementation influences rather than established determinants of programme success.

These findings informed the conceptual framework presented in Figure 2, which brings together the organisational and implementation factors identified across the evidence. The findings are broadly consistent with previous international reviews indicating that school-based physical activity promotion commonly relies on curriculum-integrated and multicomponent approaches, while implementation and sustainability are influenced by organisational and contextual conditions [14,23]. The present review adds a more specific South African perspective by highlighting how inadequate infrastructure, limited educator capacity, competing curriculum demands, and reliance on external implementation support were reported within under-resourced school settings. These observations are also broadly aligned with the wider sub-Saharan African evidence base [24], but the present review narrows the focus to implementation strategies and contextual influences within under-resourced South African primary and secondary schools. Given the heterogeneity of the included studies and the overlap between some programme publications, these similarities should be interpreted as areas of contextual convergence rather than evidence that particular implementation factors determine programme success.

### 4.1. Curriculum Integration

Curriculum integration was a recurring implementation approach across the included studies. Physical activity was incorporated into existing school structures through Physical Education, in-class activity breaks, movement-to-music sessions, Life Orientation, and curriculum-aligned academic activities [33,35,36,43,48,49,52]. For example, the KaziKidz-related interventions incorporated daily classroom activity breaks, Physical Education and dancing-to-music sessions, while the enhanced Physical Education programme was delivered within the existing school timetable [33,35,36,48,49]. Rather than indicating that curriculum integration is inherently superior, the findings suggest that embedding physical activity within established school routines may reduce the need for additional programme periods and may therefore be more compatible with settings where curriculum time and specialised facilities are constrained. However, the limited evidence on post-intervention maintenance means that compatibility during an externally supported intervention should not be equated with long-term sustainability.

### 4.2. Implementation Capacity and Stakeholder Support

Teacher preparation, motivation, workload, and access to implementation support were recurring influences on programme delivery. Several included studies provided teachers with structured lesson plans, intervention toolkits, training, or support from external personnel, including Physical Education specialists, coaches, researchers, and university students [13,33,34,35,36,38,39,48,49,52,53]. Conversely, limited teacher capacity, competing teaching responsibilities, limited instructional time, and difficulties with programme delivery were reported in several studies [38,39,42,47,48,49]. Implementation also involved the wider school and stakeholder environment, with school staff, researchers, community personnel, parents, and other external personnel contributing to programme delivery in some settings [38,39,40,44,46,51,55]. Learner engagement through games, music, dance, rewards, and other contextually appropriate activities was also reported as supporting participation [38,39,44,46,51,55].

Taken together, these findings suggest that implementation capacity is not limited to individual teacher skills but also depends on the availability of practical resources, time, organisational support, and stakeholder involvement. This may be particularly important in under-resourced schools, where limited personnel and competing educational demands can make additional programme activities difficult to accommodate. However, because several interventions relied on external personnel or research support, it remains unclear whether this level of support can be sustained in routine school practice beyond the formal intervention period.

### 4.3. Implementation Fidelity and Sustainability

Implementation fidelity was assessed inconsistently across the included studies. Although teacher training, lesson plans, intervention manuals, coaching, and other forms of implementation support were commonly reported [33,34,35,36,38,39,48,49], relatively few studies used predefined indicators to assess whether interventions were delivered as intended.

Detailed reporting of intervention adherence, dose delivered, implementation quality, and participant responsiveness was limited. For example, Arnaiz et al. [33] reported that intervention fidelity was not assessed despite differences in the level of implementation support provided across schools. This limits interpretation of intervention outcomes because differences in observed effects may reflect variation in implementation as well as differences in intervention characteristics.

Evidence regarding sustainability was similarly limited. Most interventions were relatively short in duration, and few specifically examined whether programme delivery continued beyond the intervention period [33,36,38,39,47,51].

Programme delivery was also vulnerable to contextual disruptions, including school closures, changes in the school calendar, absenteeism, weather conditions, protests, and the COVID-19 pandemic [36,39,43,47,52]. These findings suggest that the feasibility of sustaining physical activity programmes may depend not only on the intervention itself but also on whether implementation can be integrated into routine school systems and protected from competing or disruptive demands. However, the available evidence is insufficient to determine which implementation strategies are most effective for achieving long-term maintenance.

### 4.4. Implications for National Policy

The findings suggest that policy efforts to promote physical activity in under-resourced schools should address implementation capacity alongside programme provision. This may include strengthening educator professional development, protecting Physical Education and Life Orientation within the curriculum, providing affordable physical activity resources, and ensuring appropriate implementation support and monitoring.

At the system level, greater attention is needed to the organisational conditions that influence programme delivery, including teacher capacity, facilities and equipment, curriculum time, and stakeholder support. Collaboration among the Departments of Basic Education, Health, and Sport may help coordinate workforce development, infrastructure, and implementation resources, particularly in under-resourced schools.

Policy and programme development should also encourage routine reporting of implementation outcomes, including fidelity, feasibility, acceptability, adoption, and sustainability, as well as intervention outcomes. This would strengthen the evidence available to guide the translation of school-based physical activity programmes into routine educational practice.

### 4.5. Strengths and Limitations

A major strength of this review is that it synthesised implementation evidence relating specifically to school-based physical activity programmes in under-resourced South African schools. By examining implementation strategies, barriers, facilitators, and evidence gaps across diverse study designs, the review provides practical insights for researchers, educators, and policymakers seeking to strengthen implementation in resource-constrained educational settings.

Several limitations should be considered when interpreting the findings of this review. First, restricting the search to English-language publications may have excluded relevant studies published in other languages, thereby introducing language bias. Second, although comprehensive database searches and reference screening were undertaken, publication bias cannot be excluded because unpublished interventions may not have been identified. Third, substantial heterogeneity existed across study designs, intervention components, implementation duration, outcome measures, and evaluation methods, limiting direct comparison between interventions. Fourth, many included studies evaluated relatively short-term interventions, with few assessing long-term sustainability beyond one academic year. Finally, although methodological quality appraisal informed the interpretation of the evidence, the findings were synthesised narratively rather than quantitatively weighted by study quality. In addition, several publications were related through common programmes, school settings, or participant cohorts; therefore, the 24 included publications should not be interpreted as representing 24 completely independent intervention populations.

## 5. Conclusions

This scoping review identified recurring implementation strategies, barriers, facilitators, and evidence gaps relating to school-based physical activity programmes in under-resourced South African schools. Curriculum integration, educator training and support, stakeholder engagement, contextual adaptation, and appropriate resource provision were frequently reported across the included publications. However, the heterogeneity of the interventions and the limited measurement of implementation fidelity and sustainability mean that these factors should be interpreted as reported implementation considerations rather than established determinants of programme success. Figure 2, therefore, provides a conceptual synthesis of the available evidence rather than a validated implementation model.

Despite the range of implementation approaches identified, important knowledge gaps remain. The 24-publication evidence base was predominantly focused on primary school learners, with comparatively few implementation studies involving adolescents, limited long-term evaluations of programme sustainability and scalability, and uneven geographical representation across South Africa.

The presence of related publications from some programmes and samples also limits the extent to which the evidence base can be interpreted as representing independent intervention populations. Addressing these gaps through implementation science-informed research will strengthen the evidence needed to translate promising physical activity interventions into sustainable policies and routine educational practice, ultimately supporting the equitable promotion of physical activity for children and adolescents in under-resourced schools.

## Figures and Tables

**Figure 1:**
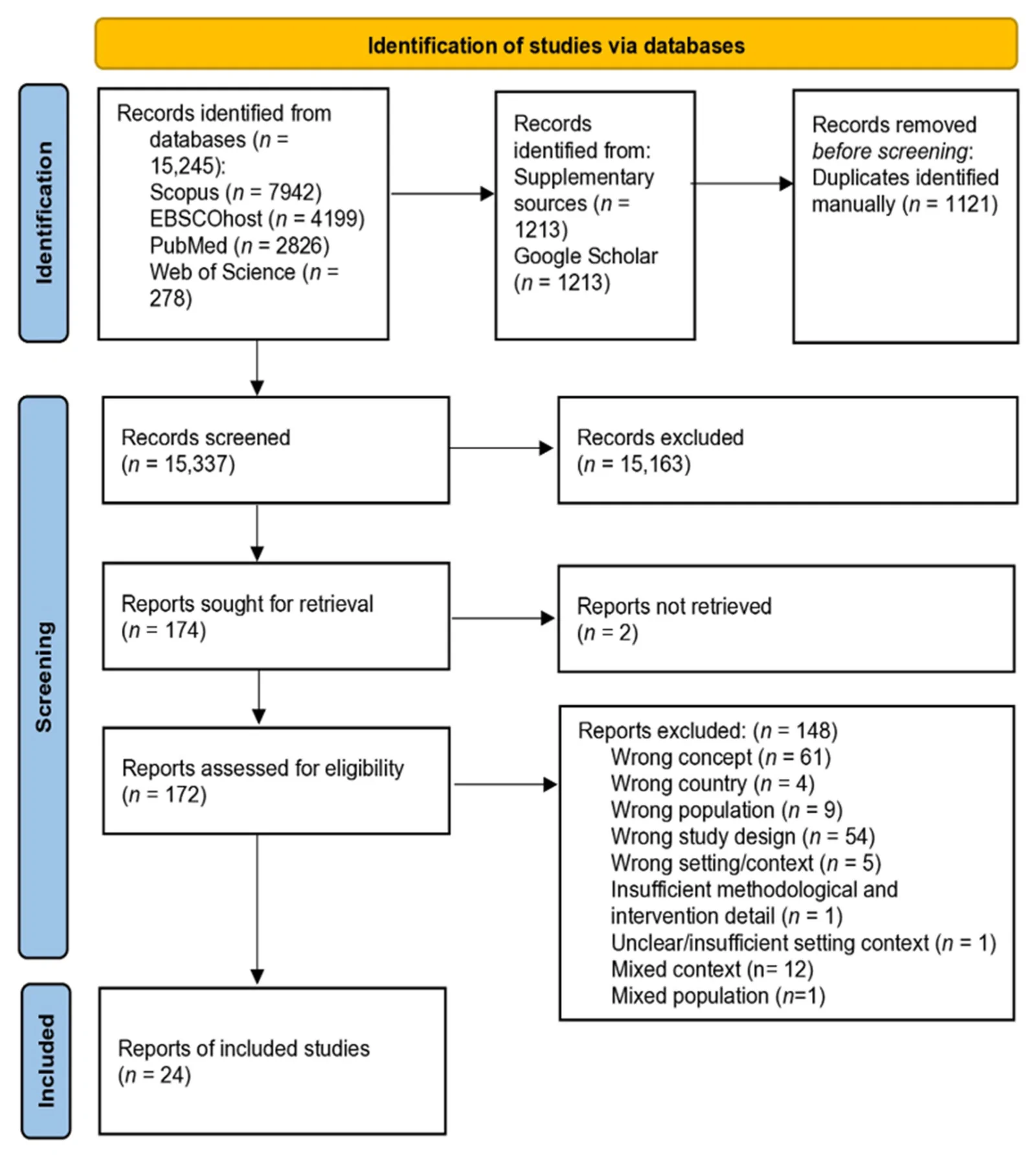
PRISMA-ScR flow diagram illustrating the study selection process, including identification, screening, eligibility, and inclusion of studies.

**Figure 2:**
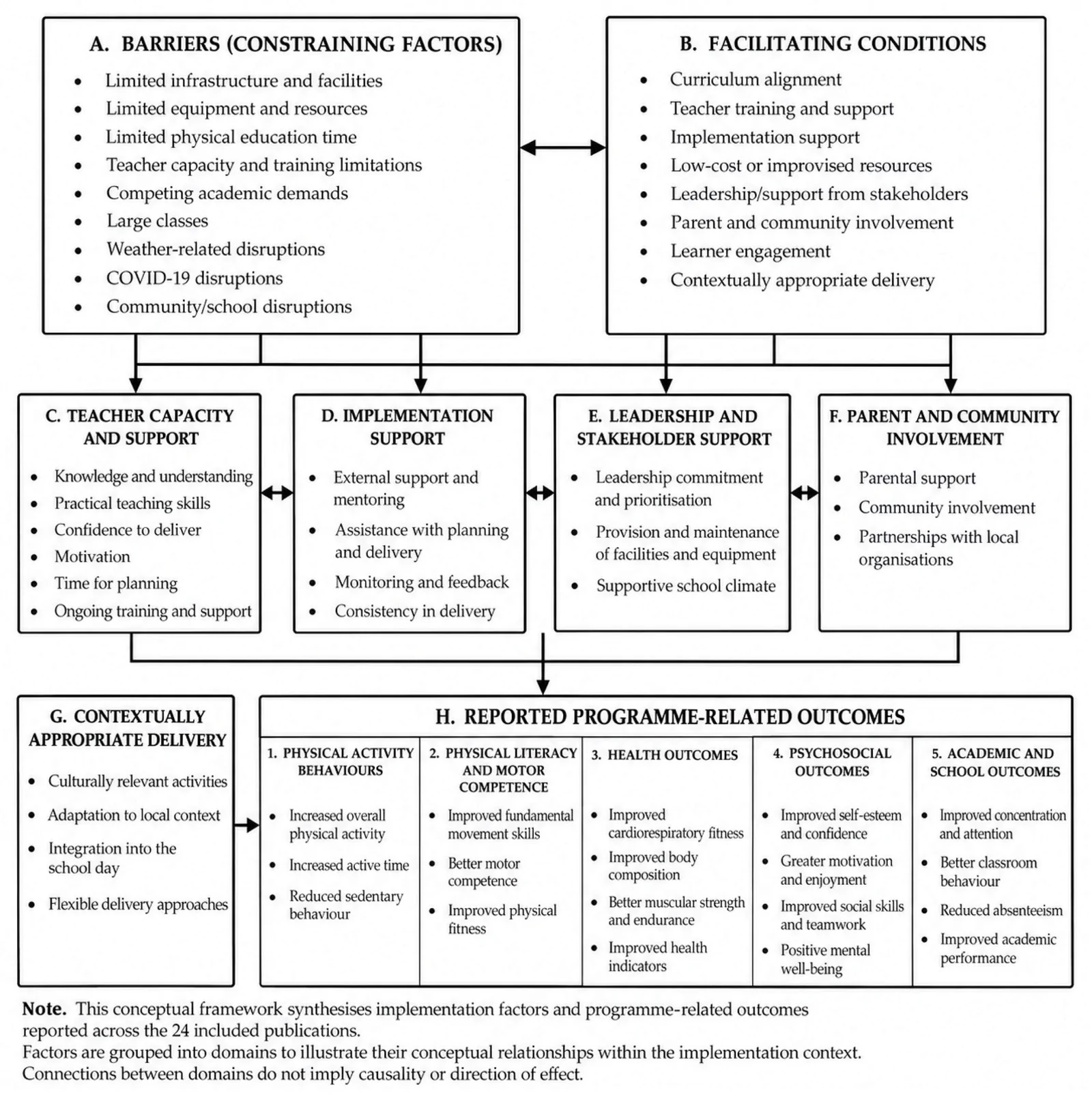
Conceptual synthesis of potential implementation influences and reported programme-related outcomes identified across the included publications. The synthesis incorporates contextual, organisational, educator, leadership, stakeholder, learner, and implementation-support factors reported in the evidence base. Arrows indicate potential interpretive relationships rather than tested causal pathways, and the figure should not be interpreted as a validated implementation model or as establishing determinants of programme success.

**Table 1:** Cross-study implementation patterns identified across included studies.

Results Theme	Core Implementation Evidence	Supporting Studies	Practical Implication for School Settings
Curriculum-integrated delivery	Physical activity interventions were frequently integrated into the school day through Physical Education, Life Orientation, classroom activity breaks, mathematics lessons, and other curriculum-aligned activities.	[13,33,34,35,36,38,39,40,42,43,44,46,48,49,52,53,54]	Integration into existing school structures may facilitate delivery where additional curriculum time is limited.
Structured motor skill focus	Several interventions incorporated fundamental movement skills, structured Physical Education, movement-based activities, active classroom strategies, or sport-based activities as core components of physical activity promotion.	[38,39,40,43,44,46,48,49,52,55]	These approaches may support development of motor competence and physical fitness, although improvements in motor skills did not consistently correspond with increased physical activity.
Teacher capacity and implementation support	Teacher training, access to implementation support, and availability of appropriately prepared Physical Education personnel varied across studies. Limited teacher preparation, competing responsibilities, large classes, and insufficient motivation were reported as challenges in some settings.	[13,33,34,35,36,38,39,40,42,43,44,46,48,49,52,53,54]	Teacher training and ongoing implementation support may facilitate consistent delivery, particularly where specialised Physical Education capacity is limited.
Infrastructure and material constraints	Limited facilities, equipment, Physical Education time, and other school resources were reported as barriers to implementation. Some interventions addressed these constraints through provision of equipment, playground adaptations, or low-cost and improvised materials.	[35,36,38,39,40,42,44,45,48,49,54]	Resource constraints may affect the feasibility and consistency of programme delivery, while appropriate equipment and adaptations may support implementation.
Contextual adaptation and stakeholder engagement	Studies incorporated contextual features such as indigenous games, traditional dance, local-language interpretation, parent or community involvement, and adaptation of activities to available school resources and conditions.	[38,39,40,43,44,46,48,49,50,51,55].	Contextual adaptation and stakeholder involvement may support acceptability and participation, although evidence regarding scalability remains limited.
Limited secondary-school evidence	The evidence base was dominated by primary-school studies, with comparatively few interventions involving secondary-school learners or adolescents.	[47,51]	Further research is needed to examine implementation of school-based physical activity interventions in secondary-school settings and among adolescents.
Fidelity and sustainability gaps	Implementation fidelity and longer-term sustainability were not consistently assessed across the included studies. Some studies reported adherence, attendance, or implementation support, while others identified short intervention periods, school-calendar constraints, COVID-19 disruption, or limited follow-up.	[13,33,36,38,39,41,43,44,45,46,47,51,53,54]	More consistent assessment of implementation fidelity, maintenance, and longer-term follow-up would strengthen understanding of whether school-based physical activity interventions can be sustained beyond the initial intervention period.

## Data Availability

The data extracted from the included studies, as well as the data collection template, are available upon reasonable request.

## Supplementary Materials

The following supporting information can be downloaded at: https://www.mdpi.com/article/10.3390/children13101289/s1, Table S1, Full data extraction of 24 included studies, presents the study characteristics and data extraction of the included studies; Table S2A. JBI Critical Appraisal of Randomized Control, Table S2B, JBI Critical Appraisal of Quasi-Experimental, Table S2C, JBI Critical Appraisal of Cohort Studies, Table S2A–C present the Joanna Briggs Institute (JBI) critical appraisal results for the included randomized controlled trials (S2A), quasi-experimental studies (S2B), and the prospective cohort study (S2C); Table S3, full search strategies for all databases, presents the complete electronic search strategies used for PubMed, Web of Science, Scopus, EBSCOhost, and Google Scholar, including search strings, search filters, and records retrieved; Table S4, Programme Clusters and Sample Overlap, presents the programme clusters and sample overlap among the included studies, identifying linked publications and, where available, shared school sites or participant samples; Table S5, PRISMA-ScR and PRISMA-S checklist, contains the Preferred Reporting Items for Systematic Reviews and Meta-Analyses Extension for Scoping Reviews (PRISMA-ScR) checklist. Table S6, 148 list of full text excluded studies, A complete list of the 148 articles excluded at the full-text stage, together with the reasons for exclusion, is provided in Supplementary Table S6.

## Abbreviations

The following abbreviations are used in this manuscript:

PA	Physical Activity
PE	Physical Education
LO	Life Orientation
FMS	Fundamental Movement Skills
CAPS	Curriculum and Assessment Policy Statement
MVPA	Moderate-to-Vigorous Physical Activity
JBI	Joanna Briggs Institute
UCDP	University Capacity Development Program
PRISMA-ScR	Preferred Reporting Items for Systematic Reviews and Meta-Analyses Extension for Scoping Reviews
PRISMA-S	Preferred Reporting Items for Systematic Reviews and Meta-Analyses for Searching
