# Supplementary material for: Implementation Strategies for Promoting Physical Activity in Under-Resourced South African Schools: A Scoping Review
Source: Children (Basel). 2026 Sep 22;13(10):1289. doi: 10.3390/children13101289 (PMC13703301; doi:10.3390/children13101289)
Supplement: Supplementary file 1 [file children-13-01289-s001.zip › Supplementary Table S6 148 list of full text excluded studies.pdf]

No.	Title	Authors	Year	Covidence #	Reason for full-text exclusion
1	"Beyond the syllabus": Morris Isaacson High School's struggle for human equality under the Apartheid education system, 1958–1990.	Glaser, C.	2021.0	#11615	Wrong concept;
2	"HealthKick": formative assessment of the health environment in low-resource primary schools in the Western Cape Province of South Africa.	de Villiers A; Steyn N.P; Draper C.E; Fourie JM; Barkhuizen G; Lombard C.J; Dalais L; Abrahams Z; Lambert E.V.	2012.0	#860	Wrong concept;
3	"Selected Papers from the 2nd Ellisras Longitudinal Study and Other Non-Communicable Diseases Studies International Conference" Special Issue Editorial.	Monyeki, K.D.	2021.0	#2516	Wrong study design;
4	A Historical Metamorphosis of Physical Education and Extra-curricular School Sport Activities in Chatsworth, Durban, South Africa (1960–2020)	Ellapen, T.J.; Paul, Y.	2021.0	#4954	Wrong concept;
5	A context-specific physical education programme for senior-phase primary and high schools in resource-poor communities in the Western Cape	Venter, T.	2022.0	#15744	Wrong concept;
6	A cost analysis of a sports-based sexual health education programme for adolescent girls in South African schools	Bango, F.; Pike, C.; Coakley, C.; Bekker, L.-G.; Cleary, S.	2022.0	#4093	Wrong concept;
7	A framework for the delivery of quality physical education and school sport in the South African public-school system	Spacey, G.B.	2021.0	#15746	Wrong concept;
8	A national study on the state and status of physical education in South African public schools	Burnett, C.	2021.0	#2972	Wrong concept;
9	A national study on the state and status of physical education in South African public schools	Burnett, C.	2020.0	#3200	Wrong concept;
10	A novel policy alignment and enhancement process to improve sustainment of school-based physical activity programming	Friday, P.J.; Beemer, L.R.; Martindale, D.; Wassmann, A.; Eisman, A.B.; Templin, T.; Zernicke, R.F.; Malinoff, L.; Schwartz, A.; Ajibewa, T.A.; Marenus, M.W.	2023.0	#15511	Wrong country;

11	A two-year history of high bone loading physical activity attenuates ethnic differences in bone strength and geometry in pre-/early pubertal children from a low-middle income country	Meiring, R.M.; Avidon, I.; Norris, S.A.; McVeigh, J.A.	2013.0	#4400	Wrong concept;
12	ADDRESSING CHALLENGES OF PE IN SOUTH AFRICAN PUBLIC SCHOOLS	Burnett, C.	2020.0	#2894	Wrong concept;
13	Acceptability and perceived fasibility of the KaziKidz health promotion intervention among educators and caregivers in schools from South Africa: a qualitative descriptive study.	Arnaiz P; Bergman MM; Seelig H; Adams L; Dolley D; Gerber M; Joubert N; Nqweniso S; Steinmann P; Utzinger J; Randt RD; Walter C; Pühse U; Müller I.	2024.0	#2064	Wrong population;
14	Active school transport among children from Canada, Colombia, Finland, South Africa, and the United States: A tale of two journeys	González, S.A.; Sarmiento, O.L.; Lemoine, P.D.; Larouche, R.; Meisel, J.D.; Tremblay, M.S.; Naranjo, M.; Broyles, S.T.; Fogelholm, M.; Holguin, G.A.; Lambert, E.V.; Katzmarzyk, P.T.	2020.0	#3263	Wrong concept;
15	Adolescent health and well-being in sub-Saharan Africa: Strengthening knowledge base and research capacity through a collaborative multi-country school-based study	Shinde, Sachin; Noor, Ramadhani Abdallah; Mwanyika-Sando, Mary; Moshabela, Mosa; Tadesse, Amare W.; Sherfi, Huda; Vandormael, Alain; Young, Tara; Tinkasimile, Amani; Drysdale, Roisin; Baernighausen, Till; Sharma, Deepika; Fawzi, Wafaie W.	2025.0	#2958	Wrong setting/context;
16	Adolescent levers for a diet and physical activity intervention across socioecological levels in Kenya, South Africa, Cameroon, and Jamaica: Mixed methods study protocol	Odunitan-Wayas, F.A.; Wadende, P.; Mogo, E.R.I.; Brugulat-Panés, A.; Micklesfield, L.K.; Govia, I.; Mapa-Tassou, C.; Mukoma, G.; Smith, J.A.; Motlhalhedi, M.; Wasnyo, Y.; Were, V.; Assah, F.; Okop, K.J.; Norris, S.A.; Obonyo, C.; Mbanya, J.C.; Tulloch-Reid, M.K.; King, A.C.; Lambert, E.V.; Oni, T.	2021.0	#5501	Wrong study design;

17	An Exploration of Health-Related Lifestyle Behaviours of Foundation Phase Learners : A Socioecological Perspective	Manyaka, M.	2022.0	#2899	Wrong study design;
18	An analysis of family-school collaboration in preventing adolescent violence in urban secondary schools	Bender, C.G.; Emslie, A.	2010.0	#5574	Wrong concept;
19	An investigation into the social factors that influence sport participation: a case of gymnastics in the Western Cape	Lucas, W.C.	2015.0	#16376	Wrong concept;
20	Association between objectively determined physical activity levels and body composition in 6–8-year-old children from a black south african population: Bc–it study	Sedumedi, C.M.; Janssen, X.; Reilly, J.J.; Kruger, H.S.; Monyeki, M.A.	2021.0	#3330	Wrong study design;
21	Association between physical activity, cardiorespiratory fitness and clustered cardiovascular risk in South African children from disadvantaged communities: Results from a cross-sectional study	Müller, I.; Walter, C.; Du Randt, R.; Aerts, A.; Adams, L.; Degen, J.; Gall, S.; Joubert, N.; Nqweniso, S.; Des Rosiers, S.; Smith, D.; Seelig, H.; Steinmann, P.; Wadhwani, C.; Probst-Hensch, N.; Utzinger, J.; Pühse, U.; Gerber, M.	2020.0	#3177	Wrong study design;
22	Association of Perceived Neighbourhood Walkability with Self-Reported Physical Activity and Body Mass Index in South African Adolescents	Wayas, F.A.; Smith, J.A.; Lambert, E.V.; Guthrie-Dixon, N.; Wasnyo, Y.; West, S.; Oni, T.; Foley, L.	2023.0	#3565	Wrong concept;
23	Association of accelerometry-based and self-reported physical activity with cardiovascular risk in South African children	Arnaiz, P.; Guntlisbergen, F.; Infanger, D.; Gerber, M.; Adams, L.; Dolley, D.; Joubert, N.; Nienaber, M.; Nqweniso, S.; du Randt, R.; Steinmann, P.; Utzinger, J.; Walter, C.; Pühse, U.; Müller, I.	2023.0	#3424	Wrong concept;
24	Associations between household socioeconomic status, car ownership, physical activity, and cardiorespiratory fitness in South African primary schoolchildren living in marginalized communities	Gerber, M.; Lang, C.; Beckmann, J.; Degen, J.; du Randt, R.; Gall, S.; Long, K.Z.; Müller, I.; Nienaber, M.; Steinmann, P.; Pühse, U.; Utzinger, J.; Nqweniso, S.; Walter, C.	2021.0	#3402	Wrong study design;

25	Associations of MC3R polymorphisms with physical activity in South African adolescents	Yako, Y.Y.; Hassan, M.S.; Erasmus, R.T.; Van Der Merwe, L.; Van Rensburg, S.J.; Matsha, T.E.	2013.0	#5011	Wrong concept;
26	Beyond the reach of FIFA: football and community ‘development’ in rural South Africa, towards a politics of inclusion and sustainability	Giampiccoli, A.; Nauright, J.	2019.0	#4345	Wrong concept;
27	Body mass index and movement behaviors among schoolchildren from 13 countries across a continuum of human development indices: A multinational cross-sectional study.	Manyanga, T.; Barnes, J.D.; Chaput, J.-P.; Guerrero, M.; Katzmarzyk, P.T.; Mire, E.F.; Prista, A.; Tremblay, M.S.; ISCOLE Research Group.	2020.0	#15089	Wrong concept;
28	Challenges facing life skills and life orientation subject advisors in implementation of physical education	Stroebel, L.C.E.; Hay, J.; Bloemhoff, H.J.	2018.0	#3220	Wrong concept;
29	Clustered cardiovascular disease risk among children aged 8-13 years from lower socioeconomic schools in Gqeberha, South Africa	Dolley, D.; Walter, C.; Du Randt, R.; Pühse, U.; Bosma, J.; Aerts, A.; Adams, L.; Arnaiz, P.; Degen, J.; Gall, S.; Joubert, N.; Müller, I.; Nienaber, M.; Nqweniso, F.; Seelig, H.; Steinmann, P.; Utzinger, J.; Gerber, M.	2022.0	#3757	Wrong study design;
30	Community mapping and creating safe spaces for physical activity in a South African context of relative poverty	Oosthuizen, Y.; Burnett, C.	2019.0	#3626	Wrong study design;
31	Comparing Adolescents at Risk and Not at Risk of NonCommunicable Disease in Terms of Body Composition and Physical Activity in the Eastern Cape, South Africa	Mvula, S.; van Gent, M.M.; van Niekerk, R.L.	2024.0	#4592	Wrong concept;
32	Describing a Design Thinking Methodology to Develop Sustainable Physical Activity and Nutrition Interventions in Low Resourced Settings	Botha-Ravyse, C.; Crichton, S.; Moss, S.J.; Hanekom, S.M.	2018.0	#5683	Wrong concept;
33	Developing Programmes to Promote Participation in Sport among Adolescents with Disabilities: Perceptions Expressed by a Group of South African Adolescents with Cerebral Palsy.	Bantjes, J.; Swartz, L.; Conchar, L.; Derman, W.	2015.0	#11759	Wrong study design;

34	Developing intervention strategies to optimise body composition in early childhood in South Africa	Draper, C.E.; Tomaz, S.A.; Stone, M.; Hinkley, T.; Jones, R.A.; Louw, J.; Twine, R.; Kahn, K.; Norris, S.A.	2017.0	#3265	Wrong study design;
35	Did HealthKick, a randomised controlled trial primary school nutrition intervention improve dietary quality of children in low-income settings in South Africa?	Steyn, N.P.; de Villiers, A.; Gwebushe, N.; Draper, C.E.; Hill, J.; de Waal, M.; Dalais, L.; Abrahams, Z.; Lombard, C.; Lambert, E.V.	2015.0	#2953	Wrong concept;
36	Disease, activity and schoolchildren's health (DASH) in Port Elizabeth, South Africa: A study protocol Health behaviour, health promotion and society	Yap, P.; Müller, I.; Walter, C.; Seelig, H.; Gerber, M.; Steinmann, P.; Damons, B.P.; Smith, D.; Gall, S.; Bänninger, D.; Hager, T.; Htun, N.S.N.; Steenkamp, L.; Gresse, A.; Probst-Hensch, N.; Utzinger, J.; Du Randt, R.; Pühse, U.	2015.0	#3382	Wrong study design;
37	Effects of a School-Based Health Intervention Program in Marginalized Communities of Port Elizabeth, South Africa (the KaziBantu Study): Protocol for a Randomized Controlled Trial.	Müller, I.; Smith, D.; Adams, L.; Aerts, A.; Damons, B.P.; Degen, J.; Gall, S.; Gani, Z.; Gerber, M.; Gresse, A.; van Greunen, D.; Joubert, N.; Marais, T.; Nqweniso, S.; Probst-Hensch, N.; du Randt, R.; Seelig, H.; Steinmann, P.; Utzinger, J.; Wadhwani, C.; Walter, C.; Pühse, U.	2019.0	#2528	Wrong study design;
38	Effects of school-based physical activity and multi-micronutrient supplementation intervention on growth, health and well-being of schoolchildren in three African countries: The KaziAfya cluster randomised controlled trial protocol with a 2 × 2 factorial	Gerber, M.; Ayekoé, S.A.; Beckmann, J.; Bonfoh, B.; Coulibaly, J.T.; Daouda, D.; Du Randt, R.; Finda, L.; Gall, S.; Mollel, G.J.; Lang, C.; Long, K.Z.; Ludyga, S.; Masanja, H.; Müller, I.; Nqweniso, S.; Okumu, F.; Probst-Hensch, N.; Pühse, U.; Steinmann, P.; Traoré, S.G.; Walter, C.; Utzinger, J.	2020.0	#4036	Wrong study design;
39	Evaluation of Physical Activity Interventions	McGoey, TN.	2020.0	#15723	Wrong study design;

40	Examining the Association between Sports Participation and Mental Health of Adolescents	Khan, A.; Ahmed, K.R.; Hidajat, T.; Kolbe-Alexander, T.; Edwards, E.J.	2022.0	#4000	Wrong country;
41	Examining the Perceived Benefits of Structured and Unstructured Physical Education Lessons among high School Learners in Cape Town	Johannes, C.; Titus, S.; Young, M.	2025.0	#3154	Wrong study design;
42	Experiences of sport coordinators in a physical education professional development programme	Zeller, D.; Roux, C.J.	2020.0	#15908	Wrong concept;
43	FOUNDATION PHASE TEACHERS' EXPERIENCES OF PHYSICAL EDUCATION IN LOW-RESOURCE SCHOOLS: IMPLICATIONS FOR CONTINUING PROFESSIONAL DEVELOPMENT	Kahts-Kramer, S.A.; DU RANDT, R.; Wood, L.	2022.0	#3133	Wrong concept;
44	Factors Influencing Break-Time Physical Activity of South African Primary School Learners From Low-Income Communities.	Uys, M.; Draper, C.E.; Hendricks, S.; de Villiers, A.; Fourie, J.; Steyn, N.; Lambert, E.V.	2015.0	#361	Wrong study design;
45	Factors Influencing Physical Activity Participation among Secondary School Learners in Hlanganani Rural Area of Limpopo Province, South Africa	Kubayi, N. A.	2021.0	#2904	Wrong concept;
46	From policy to practice for school sport: Lessons from South Africa	Burnett, C.	2020.0	#3712	Wrong concept;
47	Guidelines for action learning as professional development to transform Physical Education in low resourced primary schools in South Africa	Kahts-Kramer, S.A.; Wood, L.	2023.0	#3322	Wrong concept;
48	Health promoting schools: Consensus, strategies, and potential	Macnab, A.J.; Gagnon, F.A.; Stewart, D.	2014.0	#3551	Wrong study design;
49	Health promotion intervention in at-risk South African primary schools	Shaik Mahomed, R.; Etokabeka, E.	2025.0	#3503	Wrong concept;
50	HealthKick: a nutrition and physical activity intervention for primary schools in low-income settings.	Draper, C.E.; de Villiers, A.; Lambert, E.V.; Fourie, J.; Hill, J.; Dalais, L.; Abrahams, Z.; Steyn, N.P.	2010.0	#281	Wrong study design;

51	High bmi and low muscular fitness predict low motor competence in school-aged children living in low-resourced areas	Verbecque, E.; Coetzee, D.; Ferguson, G.; Smits-Engelsman, B.	2021.0	#3518	Wrong study design;
52	Indigenous games and physical activity for preventing non-communicable diseases in African communities: a public health perspective	Mathunjwa, M.L.; Ndlovu, P.B.; Mazibuko, S.; Mukoma, G.G.; Avramov, D.	2025.0	#3212	Wrong study design;
53	Intervention to optimise body mass index in adolescents and address the triple burden of malnutrition—the Ntshembo (Hope) trial in rural and urban South Africa study: a study protocol for a randomised controlled trial	Norris, S.A.; Micklesfield, L.K.; Christofides, N.J.; Crouch, S.H.; Mathatha, D.E.; Desmond, C.; Sharp, S.J.; Ong, K.K.; Tollman, S.M.; Kahn, K.	2026.0	#4861	Wrong study design;
54	KaziBantu 'healthy schools for healthy communities' - A holistic approach to enhance health literacy and physical activity in primary schools from low-resourced settings in South Africa.	Arnaiz, P.; Müller, I.; Dolley, D.; Adams, L.; Degen, J.; Joubert, N.; Nqweniso, S.; Gerber, M.; du Randt, R.; Walter, C.; Pühse, U.		#14221	Insufficient methodological and intervention detail;
55	Key findings of a national study on school sport and physical education in South African public schools	Burnett, C.	2020.0	#3941	Wrong concept;
56	Knowledge of students attending a high school in pretoria, South Africa, on diet, nutrition and exercise	Letlape, S.V.; Mokwena, K.; Oguntibeju, O.	2010.0	#4644	Wrong concept;
57	Leisure Constraints for Adolescents in Cape Town, South Africa: A Qualitative Study	Palen, L.A.; Patrick, M.E.; Gleeson, S.L.; Caldwell, L.L.; Smith, E.A.; Wegner, L.; Flisher, A.J.	2010.0	#2943	Wrong study design;
58	Live in the moment, educate for life: Lessons for life-long participation in structured physical activity	Sumption, B.; Burnett, C.	2021.0	#3445	Wrong study design;
59	Low levels of physical activity in female adolescents cause overweight and obesity: Are our schools failing our children?	Mokabane, M.N.; Mashao, M.M.; van Staden, M.; Potgieter, M.J.; Potgieter, A.	2014.0	#6372	Wrong concept;
60	Measuring physical activity in South African grade 2 and 3 learners: A self-report questionnaire versus pedometer testing	Malan, G.F.; Nolte, K.	2017.0	#3790	Wrong concept;

61	Moderate-to-Vigorous Physical Activity Is Associated With Cardiorespiratory Fitness Among Primary Schoolchildren Living in Côte d'Ivoire, South Africa, and Tanzania	Gerber, M.; Ayekoé, S.A.; Beckmann, J.; Bonfoh, B.; Kouassi, K.B.; Gba, B.C.; Traoré, S.G.; Coulibaly, J.T.; Daouda, D.; du Randt, R.; Finda, M.F.; Minja, E.G.; Gall, S.; Mollel, G.J.; Lang, C.; Long, K.Z.; Masanja, H.; Müller, I.; Nqweniso, S.; Okumu, F.O.; Probst-Hensch, N.; Pühse, U.; Steinmann, P.; Walter, C.; Utzinger, J.	2021.0	#3623	Wrong concept;
62	More than a Sporting Chance? Appraising the sport for development legacy of the 2010 FIFA World Cup.	Cornelissen, S.	2011.0	#12161	Wrong study design;
63	Move with Me: Co-designing a Tangible User Interface to Promote Physical Activity among Rural South African Children	Till, S.C.; Wilson, T.; Dewlok, M.; Verdezoto, N.; Densmore, M.	2025.0	#4244	Wrong concept;
64	Needs and challenges of foundation phase life skills teachers in delivering physical education: Jack of all trades and master of none?	Stroebel, L.C.E.; Hay, J.; Bloemhoff, H.J.	2017.0	#3585	Wrong concept;
65	Nutrition and physical activity randomized control trial in child care centers improves knowledge, policies, and children's body mass index	Alkon, A.; Crowley, A.A.; Neelon, S.E.; Hill, S.; Pan, Y.; Nguyen, V.; Rose, R.; Savage, E.; Forestieri, N.; Shipman, L.; Kotch, J.B.	2014.0	#15389	Wrong country;
66	Nyakaza-Move-for-Health: A Culturally Tailored Physical Activity Intervention for Adolescents in South Africa Using the Intervention Mapping Protocol.	Zimu, P.M.; Van Heerden, H.J.; Grace, J.M.	2024.0	#1600	Wrong concept;
67	Objectively determined habitual physical activity in South African adolescents: The PAHL study	Wushe, S.N.; Moss, S.J.; Monyeki, M.A.	2014.0	#4079	Wrong concept;
68	Opportunities and challenges facing NGOs using sport as a vehicle for development in post-apartheid South Africa	Sanders, B.; Phillips, J.; Vanreusel, B.	2014.0	#4016	Wrong study design;
69	Osteogenic effects of a physical activity intervention in South African black children	Meiring, R.M.; Micklesfield, L.K.; Avidon, I.; McVeigh, J.A.	2014.0	#5118	Wrong setting/context;

70	Perceived benefits of and barriers to exercise participation among secondary school students	Kubayi, N.A.; Surujlal, J.	2014.0	#4072	Wrong concept;
71	Physical Activity, Sedentary Behaviour, Weight Status, and Body Composition among South African Primary Schoolchildren	Gerber, M.; Lang, C.; Beckmann, J.; du Randt, R.; Long, K.Z.; Müller, I.; Nienaber, M.; Probst-Hensch, N.; Steinmann, P.; Pühse, U.; Utzinger, J.; Nqweniso, S.; Walter, C.	2022.0	#4507	Wrong study design;
72	Physical Education delivery in the intermediate schooling phase in South African public schools	Swanepoel, C.D.; Roux, C.J.	2024.0	#3394	Wrong study design;
73	Physical activity advocacy and promotion: The South African experience.	Kolbe-Alexander, T. L.; Bull, F.; Lambert, E. V.	2012.0	#11963	Wrong concept;
74	Physical activity and dual disease burden among South African primary schoolchildren from disadvantaged neighbourhoods	Gerber, M.; Müller, I.; Walter, C.; du Randt, R.; Adams, L.; Gall, S.; Joubert, N.; Nqweniso, S.; Smith, D.; Steinmann, P.; Probst-Hensch, N.; Utzinger, J.; Pühse, U.	2018.0	#3547	Wrong study design;
75	Physical activity and health-related quality of life among schoolchildren from disadvantaged neighbourhoods in Port Elizabeth, South Africa	Salvini, M.; Gall, S.; Müller, I.; Walter, C.; du Randt, R.; Steinmann, P.; Utzinger, J.; Pühse, U.; Gerber, M.	2018.0	#3502	Wrong study design;
76	Physical activity and sedentary behavior among adolescents in rural South Africa: levels, patterns and correlates.	Micklesfield, L.K.; Pedro, T.M.; Kahn, K.; Kinsman, J.; Pettifor, J.M.; Tollman, S.; Norris, S.A.	2014.0	#580	Wrong concept;
77	Physical activity and sedentary behavior in an ethnically diverse group of South African school children	McVeigh, J.; Meiring, R.	2014.0	#4515	Wrong study design;
78	Physical activity during school in urban minority kindergarten and first-grade students.	Reznik, M.; Wylie-Rosett, J.; Kim, M.; Ozuah, P.O.	2013.0	#219	Wrong setting/context;
79	Physical activity in early childhood education and care settings in a low-income, rural South African community: an observational study.	Tomaz, S.A.; Jones, R.A.; Hinkley, T.; Twine, R.; Kahn, K.; Norris, S.A.; Draper, C.E.	2019.0	#321	Wrong study design;
80	Physical activity of children from a small rural town, South Africa	Minnaar, E.; Grant, C.C.; Fletcher, L.	2016.0	#4245	Wrong study design;

81	Physical activity promotion	Ainsworth, B.E.; Ananian, C.D.	2020.0	#15467	Wrong concept;
82	Physical fitness of South African primary school children, 6 to 13 years of age: Discovery Vitality health of the nation study	Armstrong, M.E.G.; Lambert, E.V.; Lambert, M.I.	2011.0	#3446	Wrong concept;
83	Physical fitness profile of primary school learners in the Eastern Cape province of South Africa.	Gomwe, H.; Phiri, L.; Marange, C.S.	2024.0	#2584	Wrong study design;
84	Physics activity and sedentary behaviour of primary school learners in the Eastern Cape province of South Africa	Gomwe, H.; Seekoe, E.; Lyoka, P.; Marange, C.S.; Mafa, D.	2022.0	#2933	Wrong study design;
85	Positive Physical Education: Teaching Well-Being Skills Through Sport	Fung, D.	2023.0	#15896	Wrong concept;
86	Preschool teachers' understanding and use of supplementary activities to teach Physical Education	Veldsman, A.; du Preez, H.	2023.0	#15907	Wrong concept;
87	Prevention of Type 2 Diabetes (T2DM) in South Africa-a Focus on Facilitating Physical Activity Behaviour Change in Peri-Urban Youth	Anderson, C.; Jansen van Vuuren, A.; Learmonth, D.	2013.0	#15637	Wrong study design;
88	Primary school learners’ movement during class time: perceptions of educators in the Western Cape, South Africa	Fisher, D.; Louw, Q.	2023.0	#3351	Wrong population;
89	Professional development for physical education teachers: A participatory approach to identifying learning needs	Kahts-Kramer, S.; Wood, L.	2023.0	#3214	Wrong concept;
90	Promoting healthy lifestyle behaviour through the life-orientation curriculum: Teachers’ perceptions of the HealthKick intervention	Hill, J.; Draper, C.E.; de Villiers, A.; Fourie, J.M.; Mohamed, S.; Parker, W.; Steyn, N.	2015.0	#3398	Wrong population;
91	Promoting physical activity through primary health care: a toolkit	World Health Organization	2021.0	#15258	Wrong setting/context;
92	Recommendations to Enhance Parental Involvement and Adolescent Participation in Physical Activity	Cozett, C.; Roman, N.V.	2022.0	#3586	Wrong concept;
93	Relationship between factors of neuromotor fitness and children's indigenous games: Linkage with fundamental motor skills	Lyoka, P.A.	2011.0	#3395	Wrong study design;

94	Relationship between physical activity and risk factors of body weight disorders among south African primary school children	Moselakgomo, V.K.; Monyeki, M.A.; Toriola, A.L.	2015.0	#3689	Wrong study design;
95	Relationships between outdoor time, physical activity, sedentary time, and body mass index in children: A 12-country study	Larouche, R.; Barreira, T.V.; Hu, G.; Maia, J.; Sarmiento, O.L.; Katzmarzyk, P.T.; Mire, E.F.; Chaput, J.-P.; Lambert, E.V.; Olds, T.; Standage, M.; Belanger, K.; Fogelholm, M.; Maher, C.; Onywera, V.; Tudor-Locke, C.; Tremblay, M.S.	2019.0	#3192	Wrong concept;
96	Relevance of Olympism education and sport-for-development programmes in South African schools	Burnett, C.	2016.0	#3483	Wrong concept;
97	Results From South Africa's 2022 Healthy Active Kids' Report Card on Physical Activity, Body Composition Proxies, and Nutritional Status in Children and Adolescents	Nyawose, Z.Z., Naidoo, R., Christie, C., Bassett, S., Coetzee, D., vanGent, M., Monyeki, A., Gradidge, P., Janse van Rensburg, C., Cozett, C., Young, M., Slemming, S., Morrow, L., Pienaar,A., Krog, S., Walter, S., Kholvadia, A., De Milander, M., Naidoo, N. and Lambert, E.V.	2024.0	#15624	Wrong study design;
98	Results from South Africa's 2014 report card on physical activity for children and youth	Draper, C.; Basset, S.; de Villiers, A.; Lambert, E.V.; Uys, M.; Bartels, C.; Blomkamp, Y.; Micklesfield, L.; Kruger, S.; Puoane, T.; Naidoo, R.; Dugmore, H.; Walters, C.; Naidoo, N.; Bacon, J.; McQuaide, K.; Josephs, L.; Christie, C.	2014.0	#3149	Wrong study design;
99	Results from South Africa's 2016 report card on physical activity for children and youth	Uys, M.; Bassett, S.; Draper, C.E.; Micklesfield, L.K.; Monyeki, A.; de Villiers, A.; Lambert, E.V.	2016.0	#3297	Wrong study design;

100	Results from the healthy active kids south Africa 2018 report card	Draper, C.E.; Tomaz, S.A.; Bassett, S.H.; Harbron, J.; Kruger, H.S.; Micklesfield, L.K.; Monyeki, A.; Lambert, E.V.	2019.0	#3516	Wrong study design;
101	School communities' expectations of a school-based health promotion intervention in South Africa	Versfeld, J.; Ferreira, R.; Botha, K.	2026.0	#3299	Wrong study design;
102	School environment, socioeconomic status and weight of children in Bloemfontein, South Africa	Meko, L.N.M.; Slabber-Stretch, M.; Walsh, C.M.; Kruger, S.H.; Nel, M.	2015.0	#4839	Wrong concept;
103	School health guidelines to promote healthy eating and physical activity.	Centres for Disease Control and Prevention (CDC)	2011.0	#34	Wrong country;
104	Skipping rope and pamphlet intervention to promote physical activity among young adolescents in South Africa: study protocol for a randomised controlled trial	Ferrero, E.; Ncayiyana, J.; Machanyangwa, S.; Partap, U.; Shinde, S.; Bajaria, S.; Sivewright, N.; Neumann, C.; Sando, M.M.; Laxy, M.; Bärnighausen, T.; Fawzi, W.; Burns, J.	2026.0	#3680	Wrong study design;
105	Sotho-Tswana Games: Informal Learning Promoting Children's Well-Being in South Africa	Motsamayi, M.F.; Dlamini, P.	2024.0	#5719	Wrong study design;
106	Sport Funding of Schools in Low Socio- Economic Communities in Gauteng	Phaswana, E.N.	2018.0	#2929	Wrong concept;
107	Sport development in rural schools of Lephalale in Limpopo province: Barriers and facilitators.	Letshokotla, T.M.; Maleka, D.; Galantino, M.L.; Nkuna, R.	2024.0	#2382	Wrong study design;
108	Stakeholder Perceptions on the State and Status of Public High School Sport in Umgeni Local Municipality	Hlela, PN.	2023.0	#3060	Wrong concept;
109	Striving for equity: Life orientation resources in South African high schools	van Zyl, A.; Webb, E.M.; Wolvaardt, J.E.	2021.0	#2832	Wrong study design;
110	Sustainability of a school-based health intervention for prevention of non-communicable diseases in marginalised communities: protocol for a mixed-methods cohort study.	Arnaiz, P.; Adams, L.; Müller, I.; Gerber, M.; Walter, C.; du Randt, R.; Steinmann, P.; Bergman, M.M.; Seelig, H.; van Greunen, D.; Utzinger, J.; Pühse, U.	2021.0	#1993	Wrong study design;

111	Teachers' perceptions of physical education for children with Fetal Alcohol Spectrum Disorder at a special needs primary school within the Cape Winelands District	Lottering, A.	2025.0	#15760	Wrong population;
112	Teachers’ experiences of indigenous games in the early grades	Hadebe-Ndlovu, B.N.	2022.0	#5406	Wrong population;
113	Teenage pregnancy rates and associations with other health risk behaviours: A three-wave cross-sectional study among South African school-going adolescents	Jonas, K.; Crutzen, R.; Van Den Borne, B.; Sewpaul, R.; Reddy, P.	2016.0	#3871	Wrong concept;
114	The Food and Nutrition Environment at Secondary Schools in the Eastern Cape, South Africa as Reported by Learners.	Okeyo, A.P.; Seekoe, E.; de Villiers, A.; Faber, M.; Nel, J.H.; Steyn, N.P.	2020.0	#1903	Wrong concept;
115	The Professional Development of Non-Specialist Physical Education Teachers	Visagie, D.A.	2016.0	#2851	Wrong concept;
116	The Relationship Between School Grade and Barriers to Participation in Sport for Adolescent Learners: A South African Case Study	Klaasen, R.J.; van Gent, M.; Noorbhai, H.	2024.0	#3504	Wrong concept;
117	The State and Status of Physical Education in Selected Rural Primary Schools in the Limpopo Province, South Africa	Sakala, M.	2019.0	#2960	Wrong concept;
118	The State and Status of Physical Education in the Intermediate Phase of Selected Public Schools in Gauteng, South Africa	Swanepoel, C.D.	2021.0	#2940	Wrong concept;
119	The correlation between motor proficiency and physical activity in Senior Phase learners in the Potchefstroom area	van Niekerk, L.-L.; du Toit, D.; Pienaar, A.E.	2016.0	#3507	Wrong study design;
120	The impact of a sport-for-education programme in the South African context of poverty	Burnett, C.	2014.0	#3164	Wrong population;
121	The influence of access to facilities on the physical activity level of high school pupils in Bophelong, A semi-urban area of South Africa	van den Berg, L.; Grobler, W.C.J.	2014.0	#3204	Wrong study design;
122	The leisure and sport participation patterns of high school learners in Potchefstroom	Fourie, J.; Slabbert, E.; Saayman, M.	2011.0	#3893	Wrong concept;

123	The management and implementation of School Sport Mass Participation Programme (SSMPP) in selected secondary schools at Malamulele East Education Cluster in the Limpopo Province	Mathebula, N.S.; Milondzo, K.S.	2015.0	#16377	Wrong concept;
124	The perceived benefits of structured and unstructured physical education lessons: Perspectives from selected high schools in Cape Town	Johannes, C.	2019.0	#15751	Wrong study design;
125	The role of parental involvement in adolescents' sport participation: The case study of a non-governmental organisation in the Western Cape province. South Africa	Mahlaela, A.M.	2021.0	#16374	Wrong concept;
126	The status of Physical Education in BRICS nations	Oelofse, V.; van der Merwe, N.; du Toit, D.; de Ridder, J.H.	2024.0	#16081	Wrong study design;
127	Variables Most Strongly Associated with Motor- and Health-Related Physical Fitness and Motor Skills in Five- to Eight-Year-Old Children: The BC-It and Examin Youth SA Studies	Monyeki MA, Pienaar AE, Gericke C, Gerber B.	2026.0	#2844	Wrong study design;
128	"We Just Improvise": Exploring Teachers' Perspectives on Sport Participation for Learners with Intellectual Disabilities in Rural South Africa	Meregi, A.D.; Mulibana, P.K.; Mukoma, G.G.	2025.0	#3225	Wrong population;
129	Promoting Adolescent and Youth Health Through Physical Activity Initiatives and Interventions in Sub-Saharan Africa: The ARISE-NUTRINT and DASH Initiatives	Todorovic, N.; Ranisavljev, M.; Korovljev, D.; Mauti, J.; Neumann, C.; Mboya, I.; Ferrero, E.; Ourohiré, M.; Somé, S.; Liu, S.; Shinde, S.; Noor, R.A.; Bärnighausen, T.; Ostojic, S.M.	2025.0	#15583	Wrong concept;
130	The Perceptions and Attitudes of grade 10 life Orientation teachers and school Principals towards Physical Education in the Mankweng Circuit of the Limpopo Province of South Africa	Shongwe, TM.	2022.0	#15749	Wrong study design;
131	Indigenous games as part of school-based physical activity delivery programmes: prospects for optimum implementation for primary school learners' holistic health,,	Sakala, M.	2023.0	#15246	Wrong study design;

132	A Classroom-Based Intervention for Reducing Sedentary Behaviour and Improving Spinal Health: Pragmatic Stepped-Wedge Feasibility Randomised Controlled Trial	Fisher, D.; Maart, R.; Thabane, L.; Louw, Q.	2025		Mixed Context
133	The feasibility of an early-years motor skill school-based intervention research project in a low-resource area of South Africa	van der Walt, A.J.; Africa, E.; du Toit, D.; Pienaar, A.E.	2026		Wrong population
134	Evaluation of a school-based nutrition and physical activity programme for Grade 4 learners in the Western Cape province	Jacobs, J.; de Villiers, A.; Steyn, N.P.; Draper, C.E.; Fourie, J.; Lambert, E.V.	2013		No clear context/setting
135	Effect of a three-month HOPSports Brain Breaks® intervention programme on the attitudes of Grade 6 learners towards physical activities and fitness in South Africa	Bonnema, J.; Coetzee, D.; Lennox, A.	2020		Mixed context
136	Effect of a Three-Month HOPSports Brain Breaks® Intervention Program on the Physical Fitness Levels of Grade 6-Learners in South Africa	Bonnema, J.; Coetzee, D.; Lennox, A.	2022		Mixed context
137	The Effect of a Perceptual-Motor Intervention on the Relationship Between Motor Proficiency and Letter Knowledge	Botha, K.; Africa, E.	2020		Mixed context
138	The effect of active brain-breaks during a typical school day on the in-school physical activity patterns of Grade 1 children in the Western Cape, South Africa	van Stryp, O.; Africa, E.; Kidd, M.; Duncan, M.J.	2023		Wrong setting/context
139	The effect of a learner-support intervention on perceptual motor skills of kindergarten learners from deprived environments	Loubser, A.; Pienaar, A.E.; Klopper, A.; Ellis, S.	2016		Wrong population
140	Process evaluation of a pre-adolescent transdisciplinary health intervention for intergenerational outcomes	Arthur, K.; Christofides, N.; Nelson, G.	2021		Mixed context
141	Effectiveness of a pre-adolescent intergenerational intervention to address HIV and obesity in South Africa, using a pretest-post-test design	Arthur, K.; Christofides, N.; Nelson, G.	2021		Mixed context

142	Qualitative evaluation of a physical activity-based chronic disease prevention program in a low-income, rural South African setting	Draper, C.E.; Nemutandani, S.M.; Grimsrud, A.T.; Rudolph, M.; Kolbe-Alexander, T.L.; de Kock, L.; Lambert, E.V.	2010		Mixed population
143	Impact of a South African School-based Intervention, HealthKick, on Fitness Correlates	Uys, M.; Draper, C.E.; Hendricks, S.; de Villiers, A.; Fourie, J.; Steyn, N.P.; Lambert, E.V.	2016		Mixed context
144	Cognitive-behavioural health-promotion intervention increases fruit and vegetable consumption and physical activity among South African adolescents: a cluster-randomised controlled trial	Jemmott, J.B. III; Jemmott, L.S.; O'Leary, A.; Ngwane, Z.; Icard, L.; Bellamy, S.; Jones, S.; Landis, J.R.; Heeren, G.A.; Tyler, J.C.; Makiwane, M.B.	2011		Mixed context
145	Intervention Increases Physical Activity and Healthful Diet Among South African Adolescents Over 54 Months: A Randomised Controlled Trial	Jemmott, J.B. III; Zhang, J.; Jemmott, L.S.; Icard, L.D.; Ngwane, Z.; Makiwane, M.; O'Leary, A.	2019		Mixed context
146	The 'uptake' of a sport-for-development programme in South Africa	Burnett, C.	2015		Mixed context
147	Primary School Children's Nutrition Knowledge, Self-efficacy, and Behaviour, after a three-year healthy lifestyle intervention (HealthKicK)	de Villiers, A.; Steyn, N.P.; Draper, C.E.; Hill, J.; Gwebushe, N.; Lambert, E.V.; Lombard, C.	2016		Mixed context
148	Implementation of the HealthKick intervention in primary schools in low-income settings in the Western Cape Province, South Africa: a process evaluation	de Villiers, A.; Steyn, N.P.; Draper, C.E.; Hill, J.; Dalais, L.; Fourie, J.; Lombard, C.; Barkhuizen, G.; Lambert, E.V.	2015		Mixed context
