# Supplementary material for: Implementation Strategies for Promoting Physical Activity in Under-Resourced South African Schools: A Scoping Review
Source: Children (Basel). 2026 Sep 22;13(10):1289. doi: 10.3390/children13101289 (PMC13703301; doi:10.3390/children13101289)
Supplement: Supplementary file 1 [file children-13-01289-s001.zip › Supplementary Table S1 Full data extraction of 24 included studies.pdf]

Author, Year	Study Design	Population	Setting / Context	Physical Activity Promotion Strategy (Intervention Components)	Implementation (Delivery / Duration)	Barriers	Facilitators	Key Findings
Nqweniso et al., 2025	Cluster randomized controlled trial	1,151 primary school children (561 girls, 590 boys), aged 6–12 years from Grades 1–4.	Four Quintile 3 primary schools in marginalized peri-urban communities in Gqeberha, Eastern Cape, South Africa. The study formed part of the multinational KaziAfya project.	Four intervention arms: (1) Physical activity (PA) + multi-micronutrient supplementation (MMNS); (2) PA + placebo; (3) MMNS only; and (4) Placebo (control). The PA intervention used the KaziKidz toolkit, teacher training, one 45-minute physical education lesson/week, one 45-minute moving-to-music session/week, daily classroom activity breaks, sports equipment (balls, skipping ropes, cones, bean bags), playground markings, and curriculum-aligned teaching materials based on UNESCO Quality Physical Education (QPE) principles.	Baseline assessment occurred in 2019. The first intervention lasted 12 weeks (90 minutes/week of structured PA). The programme was intended to continue over two academic years, but the second year was disrupted by the COVID-19 pandemic. Micronutrient supplementation continued when physical activity sessions could not be implemented.	Major barriers included COVID-19 school closures, inability to continue physical education during lockdowns, large class sizes, limited lesson time due to organising learners, historically under-resourced schools, implementation challenges in disadvantaged communities, participant attrition, and sustainability concerns regarding micronutrient supplementation after project completion.	Facilitators included teacher training, ongoing support from Human Movement Science graduates serving as PE coaches, provision of sports equipment and playground games, curriculum-aligned KaziKidz resources, UNESCO Quality Physical Education framework, integration into existing school timetables, and delivery within the school environment.	The intervention did not significantly increase daily moderate-to-vigorous physical activity (MVPA). MMNS improved cardiorespiratory fitness (VO_2max) and later reduced systolic and diastolic blood pressure. The combined PA + MMNS intervention reduced HbA1c, but also showed some adverse metabolic findings, including increased body fat percentage and reduced HDL cholesterol after follow-up. The 90 minutes/week of school-based PA alone was insufficient, and that more frequent, longer, and higher-intensity physical activity interventions are needed in South African disadvantaged schools.

Fuller et al., 2010	Prospective cohort study with a control group.	370 Grade 6 and Grade 7 learners from two schools.	Two schools in Khayelitsha Township, Cape Town, South Africa.	The Football for Health programme consisted of 11 sessions, each lasting 90 minutes, with 45 minutes of football skills ("Play Football") and 45 minutes of health education ("Play Fair"). Football skills included playing football, passing, heading, dribbling, shielding, defending, trapping, building fitness, shooting, goalkeeping and teamwork. Each football skill was linked to a specific health message.	The programme was implemented over 11 weeks during the school year. Twenty-three community coaches were trained and delivered the sessions during school time. Coaches worked in male–female pairs and each pair delivered the programme to approximately 20 mixed-sex learners. The intervention was approved by the Western Cape Education Department and implemented with school support.	Random allocation of individual learners was not feasible because it would have created logistical and academic problems within schools; loss to follow-up occurred, particularly in the Grade 7 intervention group at the 3-month follow-up; negatively worded questionnaire items were difficult to translate into the local language; administering questionnaires to large groups increased organisational burden; noted that measuring changes in physical activity or body mass attributable to the programme would have been difficult.	Support from the Western Cape Education Department and participating schools; trained community coaches; detailed coach training and coach manual; football-based activities linked to health education; high attendance at sessions; children reported enjoying the programme and would recommend it to friends.	Significant improvements in health knowledge among intervention participants, with knowledge retained at 3-month follow-up; over 90% of participants reported positive attitudes towards the programme; high attendance throughout the intervention; the programme demonstrated that a football-based health education programme could be successfully implemented in South African schools.

Long et al., 2022	Cluster-randomized controlled trial.	Children aged 6–12 years attending Grades 1–4 in public schools. A total of 1,304 children were enrolled at baseline.	Public schools in peri-urban neighbourhoods of Gqeberha in the Eastern Cape Province that had poor ratings in terms of national poverty tables, income levels, dependency ratios and literacy rates.	Children allocated to the physical activity (PA) arm participated in daily in-class activity breaks, one weekly 45–60-minute playful physical education lesson, and one weekly 45–60-minute "Moving to Music" lesson. The lessons were incorporated into the main school curriculum and are described in the KaziKidz toolkit.	Children were cluster-randomized by class to one of four groups. Only teachers of the PA intervention classes received the KaziKidz lesson plans. Teachers were assisted weekly by a physical education coach to assure proper implementation. Teachers administered the MMNS or placebo tablets 5 days per week. Children were assessed at baseline and 9-month follow-up (T2).	Not explicitly reported.	Teachers of the PA intervention classes received the KaziKidz Intervention Toolkit, and were assisted weekly by a physical education coach to assure proper implementation.	Promotion of physical activity was associated with reduced overall fat mass and truncal fat mass at the 9-month follow-up. Among girls, promotion of physical activity was associated with reduced fat mass and truncal fat mass. School-based physical activity programmes can reduce childhood adiposity.
Long et al., 2024	Cluster-randomized controlled trial	1,304 children aged 6–12 years attending Grades 1–4.	Public primary schools located in Quintile 3 peri-urban marginalised communities of Gqeberha, Eastern Cape Province, South Africa.	Children allocated to the physical activity (PA) arm participated in daily in-class activity breaks, weekly 45–60-minute playful physical education lessons, and one 45–60-minute dancing-to-music lesson. Teachers of the PA classes received the KaziKidz lesson plans.	Children were recruited between February and April 2019. Data were collected at baseline (T1: February–April 2019), 9 months (T2: July–September 2019) and post-intervention (T3: August–November 2021). Project	Schools closed completely from March to mid-August 2020 because of the COVID-19 pandemic, and children attended school on a rotational basis after reopening. There was a reduced follow-up, loss of children to follow-up, and the	Teachers of PA classes received the KaziKidz Intervention Toolkit, and project staff who were physical education teachers trained in implementing KaziKidz lesson plans provided guidance to classroom teachers during implementation.	PA promotion was associated with reduced fat mass, MMNS was associated with increased fat-free mass, and both interventions had indirect associations through changes in zinc concentrations. School-based public health programmes that promote physical activity and multimicronutrient supplementation offer a possible strategy for improving child well-being and long-term health through body composition improvements.

					staff who were physical education teachers trained in implementing KaziKidz lesson plans provided guidance to classroom teachers during the moving-to-music and physical education lessons. Teachers administered the MMNS and placebo tablets 5 days per week.	pandemic disrupted household food supply and reduced physical activity of homebound children.		
Arnaiz et al., 2023	Cluster-randomized controlled trial	Primary school learners in Grades 4–6 (N = 961), aged approximately 8–13 years. A follow-up cohort of 280 children at risk of noncommunicable diseases was assessed two years post-intervention.	Eight low-income primary schools located in marginalized communities around Gqeberha, Eastern Cape, South Africa.	KaziKidz school-based health promotion programme comprising: (1) Physical Education lessons, (2) Moving-to-music dance classes, and (3) Health, hygiene, and nutrition education. The intervention also included teacher toolkits, sports equipment, painted playground games, teacher workshops, and implementation support from a trained sports graduate in one school.	The intervention lasted 20 weeks (March–July 2019). The KaziKidz toolkit was delivered to Grades 4–6 by Life Orientation teachers. Two intervention schools received two 90-minute teacher workshops, and one school additionally received implementation support from a trained sports graduate from	Intervention fidelity was not assessed. Intervention schools received different levels of implementation support, with three of the four intervention schools receiving external support. The COVID-19 pandemic and subsequent school closures disrupted programme continuity and may have contributed to the loss of long-term	Ready-to-use KaziKidz toolkit; teacher workshops; provision of basic sports equipment; painted playground games; implementation support from a trained sports graduate; integration into school teaching through Life Orientation teachers.	The intervention significantly increased moderate-to-vigorous physical activity (MVPA) during school hours, particularly among physically inactive children and girls. Improvements in some cardiometabolic outcomes were observed in the short term, but these effects were not maintained at the 2-year follow-up. Schools are important settings for promoting physical activity, but structural changes are needed to achieve sustainable long-term impact in marginalized communities.

					Nelson Mandela University.	intervention effects.		
Beckmann et al., 2022	Cluster-randomized controlled trial.	Primary school children aged 6–12 years (Grades 1–4); 932 completed baseline and post-intervention assessments.	Four Quintile 3 public primary schools located in a peri-urban area of Nelson Mandela Bay, Eastern Cape, South Africa.	Children allocated to the physical activity group received two weekly 45-minute physical activity lessons (one "Moving to Music" lesson and one Physical Education lesson). The lessons were based on the KaziKidz toolkit, developed according to the South African curriculum.	The intervention was implemented between April and September 2019. Because of the June–July school holidays, the actual intervention period comprised 12 weeks. The physical education coach assisted the class teacher in implementing the physical activity lessons.	The intervention was interrupted by a three-week school holiday; frequency and intensity of the physical activity intervention may not have been sufficient; large class sizes can pose challenges to the intensity and quality of physical education lessons; lack of qualified physical education teachers at primary school level in South African public schools.	The physical education coach assisted the class teacher to implement the physical activity lessons.	No significant group × time differences were observed for cognitive performance or academic achievement. The school-based physical activity intervention showed no benefit compared with the placebo group.
Zimu et al., 2024	Quasi-experimental study.	129 adolescents from eight secondary schools. Participants were adolescents identified as insufficiently physically active (PAQ-A score <2.75); 85 were assigned to the treatment group and 44 to the control group.	Eight secondary schools in Clermont and Mpumalanga townships within the eThekwini Metropolitan Municipality, KwaZulu-Natal, South Africa. The study was conducted in resource-constrained communities.	Nyakaza Move-for-Health intervention programme, comprising (1) a social marketing campaign and (2) an after-school physical activity programme. The social marketing campaign used infographics, posters and diaries to promote physical activity. The after-school programme included structured	The intervention was delivered over 12 weeks by postgraduate students in Sport Science, trained community healthy lifestyle coordinators, school educators, and research assistants. The social marketing campaign was	Participants did not achieve the recommended physical activity threshold despite improvements. Participant retention was also identified as a challenge, and the authors recommended longer and more intensive interventions.	Promotional materials (posters, infographics, diaries, T-shirts and watches), positive feedback from facilitators, parental support, music, and enjoyment motivated participation in the programme.	The intervention significantly increased physical activity levels, reduced BMI and waist-to-hip ratio, and improved knowledge, attitudes and motivation towards physical activity. Participants reported greater awareness of the benefits of physical activity and the risks of physical inactivity.

				cardio dance classes and muscular endurance circuit training.	implemented in schools, and the after-school programme provided moderate-to-vigorous physical activity through structured cardio dance and muscular endurance circuit training.			
Pienaar et al., 2012	Longitudinal quasi-experimental controlled intervention study	309 African adolescents (158 boys and 151 girls) aged 13–17 years. The intervention group comprised 222 participants and the control group 87 participants.	Two secondary schools in a low socio-economic township area (Ikageng, Potchefstroom), North-West Province, South Africa. The schools were selected because of the high prevalence of growth retardation among children. Most of the children had very poor socio-economic backgrounds.	The intervention consisted of 20 minutes of aerobic exercise at a moderate- to high-intensity to the beat of music, 20 minutes of active participation in sports-related ball games/skills (specifically soccer and netball skills), and 20 minutes of strength and flexibility exercises.	The intervention group participated in the programme for approximately one hour, twice a week for 23 weeks during 2004. In 2005, the frequency increased to three times per week for 19 weeks, excluding the four-week July school recess. No intervention took place in 2006, although follow-up measurements were conducted.	Attendance was especially poor during the middle of 2004 because of the four-week school recess. The most important reasons for not attending were responsibilities at home and living far from school.	Not explicitly reported.	The physical activity intervention showed a stabilising effect on physical activity levels and contributed to more active choices among the intervention group of boys, especially during weekends. Participation in similar physical activity interventions is recommended for adolescent boys, with adaptation of the content for girls.

Nqweniso et al., 2020	Cluster-randomized controlled trial	853 primary school children (435 boys, 418 girls) aged 8–13 years, recruited from Grade 4 classes.	Eight Quintile 3 (disadvantaged) primary schools in Port Elizabeth (Gqeberha), Eastern Cape, South Africa. Schools were located in socioeconomically disadvantaged communities.	School-based health promotion programme consisting of physical activity, health education, deworming, and nutritional supplementation. The physical activity component formed part of the intervention, while extracurricular sport participation was also assessed as an important contributor to health outcomes.	The intervention was delivered twice: July–September 2015 and February–April 2016, with each intervention lasting 10 weeks. Measurements were conducted at baseline (February 2015) and post-intervention (May 2016).	The study was conducted only in one province, limiting generalisability. Sport participation was measured using a self-report questionnaire, which may introduce reporting bias. Information on frequency but not duration of extracurricular sport participation was collected.	The intervention integrated multiple health promotion components (physical activity, health education, deworming, and nutritional supplementation). The school setting enabled implementation among children from disadvantaged communities. Maintaining cardiorespiratory fitness and encouraging regular extracurricular sport participation were identified as important health-promoting factors.	At baseline, 18.8% of children were overweight/obese and 13.5% were hypertensive. Children with higher cardiorespiratory fitness and greater extracurricular sport participation were less likely to be overweight/obese, while higher sport participation was associated with lower odds of hypertension. Longitudinally, children who participated in the physical activity intervention were less likely to become overweight/obese, although no significant preventive effect was found for hypertension. Regular physical activity and maintaining cardiorespiratory fitness are important for preventing overweight and promoting cardiovascular health in South African schoolchildren.
Tian, du Toit & Toriola, 2017	Pretest–posttest control-group design	110 Grade 7 learners aged 12–13 years from two primary schools (experimental group = 40; control group = 70).	Two primary schools in Potchefstroom, North West Province, South Africa. The schools were situated in low socio-economic areas and had large open fields.	The intervention was an enhanced quality PE programme based on the Curriculum and Assessment Policy Statement (CAPS). It included physical fitness activities (weeks 1–6) and indigenous games (weeks 7–12). Each lesson comprised a 10-minute warm-up and introduction, 30	The programme was presented for 60 minutes once a week over 12 weeks, as prescribed in the CAPS. It was presented by four pre-service PE teachers.	PE was allocated only one hour per week in CAPS. No apparatus were available for teaching PE lessons at the schools. Teachers in the control groups had received one afternoon's PE teacher preparation programme.	The quality-enhancing components were well-trained PE teachers, homework activities, a reward system, improvised apparatus, and monitoring of the intensity of activities.	The experimental group showed statistically significant increases in moderate PA, vigorous PA and total PA, and a decrease in sedentary behaviours after the 12-week intervention, whereas no significant changes were found in the control group. The enhanced quality PE programme can be used as a valuable framework for PE implementation targeted at promoting learners' PA levels, even in the presence of restricted time allocation and

				minutes of moderate- to high-intensity aerobic exercise (such as rope skipping, aerobic dance, throwing Jukskei and playing soccer), and 20 minutes of strength and muscular endurance activities (such as sit-ups, crab soccer, ball dribbling and wheelbarrow exercises). The programme also included homework activities, a reward system, improvised apparatus, and monitoring of activity intensity.				limited teaching and learning resources.
Tian, du Toit & Toriola, 2014	Experimental pretest–posttest control-group study	110 Grade 7 learners aged 12–13 years (experimental groups, n=40; control groups, n=70) from two primary schools.	Two primary schools in Potchefstroom, North West Province, South Africa. Learners had similar socio-economic backgrounds. Both schools had large open fields, but no apparatus were available for Physical Education lessons.	The enhanced quality Physical Education programme was based on the Curriculum and Assessment Policy Statement (CAPS). Activities included physical fitness activities (weeks 1–6) and indigenous games (weeks 7–12). Each lesson consisted of a 10-minute warm-up and introduction, 30 minutes of moderate- to high-intensity aerobic	The programme was presented for 60 minutes once a week over 12 weeks, as prescribed by the CAPS. The experimental groups received the enhanced quality PE programme, while the control groups attended the usual PE classes.	No apparatus were available for Physical Education lessons at the schools. The control groups were taught by teachers who had received one afternoon's training after the reintroduction of Physical Education.	The programme emphasised well-trained PE teachers, homework activities, a reward system, improvised apparatus, and monitoring of the intensity of activities.	The intervention resulted in statistically significant improvements in sit and reach, standing broad jump, sit-ups, 20 m shuttle run, plate tapping, and 10 × 5 m shuttle run among the experimental groups, while no significant improvements were found in the control groups. The enhanced quality PE programme appears to be effective in improving physical and motor fitness among South African primary school children and provides a valuable framework for PE curriculum development and implementation.

				exercise (rope skipping, aerobic dance, throwing Jukskei and playing soccer), and 20 minutes of strength and muscular endurance activities (sit-ups, crab soccer, ball dribbling and wheelbarrow exercises). The programme emphasised five components: well-trained PE teachers, homework activities, a reward system, improvised apparatus, and monitoring of activity intensity.				
Idamokoro et al., 2024	Randomized controlled trial	70 Grade 2 learners (7–8 years old); Intervention group (n=35) and Control group (n=35).	Three Quintile 3 rural primary schools in the Raymond Mhlaba Municipality, Eastern Cape Province, South Africa.	Fundamental movement skills (FMS) programme based on the SPARK Physical Education programme and aligned with the South African CAPS curriculum. Activities targeted locomotor skills, ball skills, balance activities and aerobic activities to improve motor competence and physical activity.	The intervention was implemented for 9 weeks, twice per week, 30 minutes per session (18 sessions) during school hours. Each lesson included a 5-minute warm-up, 20-minute main activity, and 5-minute cool-down. The programme was delivered by the researcher and four trained postgraduate	The intervention lasted only 9 weeks because of the South African school calendar. No Physical Education programme or PE teachers were available at the intervention schools. Very hot and rainy weather occurred during programme delivery. After the intervention, PE periods returned to their usual use (e.g., food breaks, refreshments or	The programme achieved 97% compliance. It was delivered by trained research assistants, supported by trained interpreters, with learners working in small groups, and attendance was recorded throughout implementation.	The intervention did not improve moderate-to-vigorous physical activity (MVPA) immediately or six months after the intervention, despite significant improvements in fundamental movement skills. No positive association was found between FMS and MVPA. Movement programmes should incorporate strategies that better promote children's physical activity behaviour and recommended longer interventions with clearer emphasis on physical activity promotion.

					Human Movement Science research assistants, supported by trained interpreters. Learners were organised into small groups of five, and attendance was monitored throughout the programme.	academic catch-up), and classroom teachers did not consider themselves able or motivated to teach PE. Intervention also may have been too short to produce desirable changes in physical activity.		
Nqweniso et al., 2021	Cluster-randomized controlled trial.	898 Grade 4 children (458 boys and 440 girls) aged 8–11 years.	Eight Quintile 3 schools from socioeconomically disadvantaged schools in Port Elizabeth (Gqeberha), South Africa.	The physical activity intervention consisted of two weekly 40-minute physical education lessons, one weekly 40-minute Moving-to-Music lesson, regular in-class physical activity breaks, and adaptation of the school playground to provide a physical activity-friendly environment (painted games and physical activity stations). All physical education lessons were aligned with the prescribed physical education curriculum.	The intervention was implemented over 10 weeks in four randomly selected schools. Teachers were assisted by external physical education specialists in conducting the physical activity intervention, and Moving-to-Music lessons were conducted by trained dance students from Nelson Mandela University.	The intervention period was relatively short, lasting for only 10 weeks, because the study schedule had to take into consideration school holidays, mid-term assessments and end-of-term examinations pertinent to the schools."	Teachers were assisted by external physical education specialists, and the Moving-to-Music lessons were conducted by trained dance students from Nelson Mandela University.	The physical activity intervention (alone or combined with health education) mitigated increases in body fat in normal-weight children. In overweight/obese children, the physical activity intervention alone mitigated increases in body fat. School-based physical activity, nutrition and health and hygiene interventions can have beneficial effects on children's body composition.

Williams, 2018	Pretest–posttest intervention study	330 Grade 1–3 learners (approximately 7–9 years old) from two schools.	Two at-risk school community contexts in Pretoria, South Africa. Schools formed part of the national feeding scheme and were situated in poverty-stricken communities.	School-based health promotion intervention focusing on physical fitness, healthy eating habits, and psychosocial well-being. Weekly sessions included activities and discussions on physical fitness, healthy eating, and psychosocial well-being.	Implemented during the second half of 2015, comprising three sessions per week for six weeks. Learners participated in physical fitness activities and discussions, and handouts were distributed to parents after each session, with some activities completed at home involving parents or family members.	Not reported.	Parental involvement through take-home activities and handouts after each session.	A school-based health promotion intervention integrating physical fitness, healthy eating and psychosocial well-being was implemented over six weeks in two at-risk school communities. The study evaluated psychosocial well-being and found no overall positive effect following the intervention. Physical activity or physical fitness outcomes were not reported.
Van der Merwe et al., 2023	Randomized controlled trial	Grade 1 learners from two Quintile 3 schools in Mangaung. Baseline data were collected from 57 learners, with 44 complete datasets available for analysis (control = 18; intervention = 26). Participants were 5–7 years old.	Two Quintile 3 schools in Mangaung, Free State Province, South Africa. The schools represented low socioeconomic environments, as Quintile 1–3 schools are predominantly no-fee schools.	A South African curriculum-aligned Physical Education intervention using the KaziKidz toolkit. The intervention included perceptual motor activities aligned with the CAPS curriculum. The KaziKidz toolkit comprised three pillars: Physical Education, Moving to Music, and Health, Hygiene and Nutrition Education. Lessons followed a 40-minute structure consisting of a 10-minute warm-up, 20-	The intervention was delivered during the allocated Physical Education periods by two student biokineticists. The intervention lasted 12 weeks and included 10 intervention sessions. The control group continued with Physical Education sessions presented by their teacher.	Due to the COVID-19 pandemic, slightly fewer intervention sessions were presented than planned, and participant loss occurred during post-testing because of the earlier closing of schools for school holidays.	The KaziKidz toolkit was designed in line with the South African CAPS curriculum, providing structured, age-appropriate activities, educator manuals, lesson plans and cue cards. The intervention was presented by movement specialists (student biokineticists) during allocated	Following the intervention, the intervention group showed significantly improved visual-motor integration ($p = 0.042$) and visual perception ($p < 0.001$) compared with the control group. No significant differences were observed for motor coordination. Exposure to Physical Education including perceptual motor aspects significantly improved the visual-motor integration and visual perception of 6-year-old children, and that the KaziKidz toolkit was a successful mode of Physical Education delivery for Grade 1 learners.

				minute main activity, and 10-minute cool-down.			Physical Education periods, and no academic time was lost.	
Idamokoro et al., 2024	Two-group quasi-experimental pretest–posttest study with 6-month follow-up	Grade 2 school children aged 7–8 years (N = 93); Intervention group = 57, Control group = 36.	Rural primary schools in Raymond Mhlaba Municipality, Eastern Cape Province, South Africa.	Fundamental Movement Skills (FMS) movement programme comprising locomotor skills, ball skills, balance activities and aerobic activities. The programme was adapted from the SPARK Physical Education programme.	Implemented over 9 weeks during school hours. Learners received 18 sessions, 30 minutes per session, twice weekly. Sessions included a warm-up, aerobic activities, locomotor activities, ball skills, balance activities and a cool-down. The programme was delivered by the researcher and four trained Human Movement Science	The intervention period was limited to 9 weeks because of the available school term. Only three schools agreed to participate after recruitment, and participant attrition occurred because some learners changed schools or were absent during follow-up testing.	Learner compliance with the intervention was 97%. The programme was delivered by trained assistants, interpreters were used when necessary, learners worked in small groups, and attendance was monitored throughout the intervention.	The intervention resulted in significant immediate and sustained improvements in locomotor skills, ball skills, and the Gross Motor Index (GMI) at post-test and 6-month follow-up. A short-duration FMS intervention can improve motor competence among rural school children and recommended similar programmes to enhance FMS development in rural schools.

					postgraduate assistants, with interpreters assisting where required.			
Africa & Van Deventer, 2015	Quasi-experimental pretest–posttest study with intervention and control groups	Grade 5 learners aged 9–12 years (N = 67; Experimental = 35; Control = 32)	Quintile 2 (previously disadvantaged) primary school, Stellenbosch, Western Cape, South Africa	Structured motor skills development programme focusing on balance and bilateral coordination. Activities included dynamic and static balance, bilateral coordination, rhythmic movements and target games, developed in accordance with the CAPS curriculum.	12-week intervention implemented during the second and third school terms. The programme was delivered during Physical Education lessons, with two 30-minute sessions per week. It was implemented by the researchers, including a qualified Kinderkineticist, with assistance from the Grade 5 Physical Education teacher when available.	Not explicitly reported.	The programme was aligned with the CAPS curriculum. The programme may help empower teachers to implement the lessons independently after completion of the study.	The intervention resulted in a significant improvement in bilateral coordination, while balance showed a positive but non-significant improvement. The programme may be beneficial for primary school children, particularly those from previously disadvantaged schools.

Idamokoro et al., 2023	Two-group quasi-experimental pretest–posttest study with 6-month follow-up	Grade 2 learners aged 7–8 years from rural primary schools (N = 93; Intervention = 57; Control = 36).	Three rural primary schools in Alice, Raymond Mhlaba Municipality, Eastern Cape, South Africa.	Fundamental Movement Skills (FMS)-based movement programme designed to improve movement proficiency, body composition and fitness. The programme included locomotor skills (running, galloping, hopping, skipping, jumping and sliding), manipulative skills (striking, bouncing, dribbling, catching, kicking, overhand and underhand throwing), balance activities, and aerobic exercises (rope skipping, running, jumping jacks and traditional dance). The programme was adapted from the SPARK Physical Education programme.	Implemented over 9 weeks during school hours, comprising 18 sessions delivered twice weekly (30-minute sessions) during Physical Education. Each session included a warm-up, core movement activities and cool-down. The intervention was delivered by the researcher and four trained research assistants, with translators assisting learners who did not understand English. Learners worked in groups of five to maximise participation.	The intervention duration was limited to 9 weeks because of the school term; Physical Education time within the curriculum was minimal, affecting programme delivery; only children aged 7–8 years from one rural area participated, limiting generalisability; lack of facilities, equipment, resources and qualified Physical Education teachers may discourage meaningful implementation; the programme did not improve all body composition measures; further research should consider programme duration and activity selection.	High learner compliance (97% attendance); programme delivered during Physical Education periods; use of trained research assistants; learners worked in small groups to maximise participation; incorporation of enjoyable aerobic activities (e.g., rope skipping and traditional dance); programme adapted from the SPARK Physical Education programme; translators assisted learners when needed.	The intervention significantly improved BMI, waist circumference, and subscapular skinfold, with sustained improvements in BMI and subscapular skinfold at the 6-month follow-up. The prevalence of overweight/obesity decreased from 19.3% to 10.5% in the intervention group. A low-cost, school-based movement programme focusing on fundamental movement skills and aerobic activities can improve body composition and should be considered as a strategy to combat childhood obesity in resource-limited rural schools.

Gall et al., 2020	Cluster-randomized controlled trial.	Primary school children aged 8–13 years (N = 758) from eight disadvantaged primary schools. Intervention group: 310 learners; Control group: 448 learners.	Eight disadvantaged primary schools in the Nelson Mandela Bay District (Port Elizabeth), Eastern Cape, South Africa.	Multi-component school-based physical activity intervention comprising two 40-minute physical education lessons per week, one weekly 40-minute moving-to-music lesson, regular classroom physical activity breaks, and improvements to the school environment through installation of physical play structures (jungle gyms, monkey bars, over-and-under bars) and colourful painted playground games.	The intervention was implemented over 20 school weeks. Prior to implementation, workshops demonstrated lessons, games and classroom management techniques. Physical education lessons were delivered by class teachers supported by an experienced physical education coach, while moving-to-music sessions were conducted by Nelson Mandela University student dancers after school.	Limited exposure and intensity of the intervention; physical activity increased more in the control group than in the intervention group; implementation quality depended on teachers' motivation and skills; only one school received each intervention combination; physical activity was measured using a self-report instrument rather than objective measures.	Workshops before implementation; teacher support from an experienced physical education coach; structured lesson plans; involvement of Nelson Mandela University student dancers; improvements to the school environment through installation of play structures and playground games.	Participation in the intervention did not improve health-related quality of life (HRQoL). However, higher baseline physical activity and increases in physical activity predicted better HRQoL. Schools should provide physical activity-friendly environments and implement regular quality physical education to promote physically active lifestyles among children in disadvantaged settings.

Gall et al., 2018	Cluster randomized controlled trial.	Grade 4 learners aged 8–13 years from eight primary schools. Final sample: 663 learners (Intervention = 265; Control = 398).	Eight Quintile 3 primary schools located in disadvantaged communities in Port Elizabeth, Eastern Cape, South Africa.	Multi-component school-based physical activity intervention comprising two 45-minute physical education lessons per week, one weekly 45-minute moving-to-music lesson, regular classroom physical activity breaks, and adaptation of the school environment to create a physical activity-friendly setting.	The intervention was implemented over 20 weeks (two 10-week blocks) during official school lessons. Physical education lessons were delivered in collaboration with Life Orientation teachers, moving-to-music lessons were led by Nelson Mandela University students, and classroom activity breaks were incorporated into the school curriculum.	The intervention period was relatively short; large class sizes and a heterogeneous learner population complicated implementation; teacher compliance and adherence varied considerably; some teachers lacked motivation to be physically active; school-wide implementation prevented randomisation at class level.	Information sessions were held with principals, teachers, parents and school governing bodies before implementation; teacher workshops were conducted; physical education lessons were delivered collaboratively with Life Orientation teachers; Nelson Mandela University students delivered moving-to-music sessions; the school environment was adapted to support physical activity.	The intervention significantly maintained academic performance compared with the control group, although no significant effect was found for selective attention. School-based physical activity should be implemented as part of the school curriculum in disadvantaged schools.

Müller et al., 2019	Cluster-randomized controlled trial.	Grade 4 learners aged 9–14 years from eight disadvantaged primary schools. Complete baseline data were available for 746 children.	Eight Quintile 3 disadvantaged primary schools in Port Elizabeth, Eastern Cape, South Africa.	Multidimensional school-based physical activity intervention consisting of (i) two 40-minute physical education lessons per week; (ii) one weekly 40-minute moving-to-music lesson; (iii) regular in-class physical activity breaks incorporated into the school curriculum; and (iv) enhancement of the school environment through installation of activity stations and painted games to promote physical activity.	The intervention was implemented during two 10-week intervention periods (July–September 2015 and February–April 2016) during official school hours. Physical education lessons were delivered in collaboration with Life Orientation teachers, who were supported by a trained physical education coach for one lesson each week. Moving-to-music classes were delivered by Nelson Mandela University students. Teacher workshops and project information sessions were conducted before implementation. Physical education lessons followed	Teachers' motivation to facilitate physical education varied and was influenced by unequal class sizes, lack of formal qualifications in physical education, and the fact that physical education lessons were generally not taught regularly. The intervention was implemented in only three of the eight schools and for two 10-week periods because of logistical reasons. A longer intervention and greater involvement of school communities, volunteers and parents/guardians may be needed.	The intervention was developed in collaboration with education authorities, teachers and learners. Teacher workshops and information sessions were conducted before implementation. Teachers were supported by a trained physical education coach, and sports equipment (e.g., skipping ropes, cones, balls and beanbags) was donated to schools. The intervention was positively perceived by teachers (mean evaluation 4.2/5), and education authorities expressed interest in continuing the programme. Learners particularly enjoyed the moving-to-music classes.	Compared with the control group, learners receiving the intervention had a significantly lower increase in BMI-for-age Z-score ($p < 0.001$) and reduced increase in skinfold thickness ($p = 0.007$). No significant improvement in cardiorespiratory fitness was observed. A well-designed multidimensional school-based physical activity programme can reduce increases in cardiovascular risk factors among children in disadvantaged South African schools, but longer and more intensive interventions may be required to improve cardiorespiratory fitness.

					the CAPS curriculum, lasted 40 minutes, and included a 5-minute warm-up and cool-down. Sports equipment was donated to participating schools.			
Ferguson et al., 2015	Pretest–posttest quasi-experimental study.	Children aged 6–10 years attending Grades 1–5 in a low-income primary school. The evaluation included children with Developmental Coordination Disorder (DCD) (n = 22) and children without DCD (n = 19).	Low-income primary school in Cape Town, South Africa. The school had approximately 800 learners, limited recreational facilities, two tarred playgrounds, and Physical Education offered once a week for approximately 30 minutes.	A whole-school Health Promotion Programme (HPP) designed using the WHO Health Promoting Schools framework. The programme increased opportunities for physical activity through playground games, structured Physical Education lessons, role modelling, provision of basic equipment (e.g., skipping ropes and balls), playground adaptations, educator collaboration, health education, and referral pathways for children requiring additional support.	The HPP was implemented over 9 weeks. Undergraduate physiotherapy students were present at the school 26 hours per week. The programme was delivered to all learners in Grades 1–5 through collaboration with teachers, classroom activities, playground activities, Physical Education sessions, health promotion campaigns, educator support, and environmental modifications.	High learner-to-educator ratio (40:1); insufficient time for Physical Education; inadequate facilities; shortage of equipment; educators not qualified or experienced in teaching Physical Education; limited recreational facilities; concerns regarding community safety that restricted extracurricular physical activity opportunities.	Collaboration between physiotherapy students and educators; use of the WHO Health Promoting Schools framework; playground adaptations; provision of basic equipment; role modelling by physiotherapy students; educator support and collaboration; integration of physical activity into Physical Education; referral pathways for children requiring additional services.	Both groups improved in motor performance and physical fitness following the intervention. Children with DCD demonstrated greater improvements in motor performance, while both groups showed improvements in functional strength, aerobic fitness, and anaerobic fitness. Increasing opportunities for physical activity within the school environment may improve motor performance and fitness among children in low-income schools.

Fisher & Africa, 2023	Quasi-experimental pretest–posttest study	Grade 1 learners (N = 119), aged 6–8 years	Two Quintile 1 no-fee schools in Cape Town, Western Cape, South Africa.	Physically active academic intervention integrating physical activity into mathematics lessons. Activities included sensory integration, perceptual-motor skills, balance, directionality, laterality, coordination, spatial awareness, body awareness, strength and resistance activities, stretching, aerobic fitness, number patterns, shape and space, and measurement and data handling.	Intervention delivered over 16 weeks; 30-minute sessions twice weekly during the morning; moderate-to-vigorous intensity; delivered by the researcher and a qualified Kinderkineticist; implemented in and outside the classroom using an integrated bottom-up and top-down approach.	Limited teacher involvement because teachers had to supervise other classes; wet weather prevented some outdoor sessions; community protests prevented access to schools, resulting in approximately 8.33% of intervention time being lost.	Teacher participation and buy-in; no additional teaching time required; integrated into the existing school curriculum; no additional cost; suitable for indoor and outdoor implementation.	The intervention group showed significant improvements in mathematics performance, upper-body strength, and abdominal strength/endurance. No significant differences were found for BMI, aerobic capacity, trunk-lift, or sit-and-reach flexibility.
Africa, van Stryp & Vermaak, 2026	Pilot quasi-experimental study	118 children aged 6–8 years enrolled in Grade R and Grade 1 (experimental group = 67; control group = 51). Quintile 2, government school.	A primary school outside Stellenbosch, Western Cape, South Africa. The school was selected for logistical and practical reasons, including its proximity to the researchers and willingness to participate.	Self-designed gross motor intervention focusing on fundamental movement skills (locomotor, object-control and stability skills). Parents, caregivers and teachers received a manual, flashcards and participated in an information session. The intervention included activities targeting running, jumping, hopping, skipping, galloping, sliding,	The experimental group participated in a structured 6-week intervention, conducted once per week for 45 minutes. The control group continued normal school activities during the intervention period and received the toolkit after post-testing.	The study reported no formal power calculation because it was a pilot study; a follow-up assessment could not be conducted because of the duration of the South African school terms; the intervention could not be extended beyond six weeks; and the large number of assessors may	Parents, caregivers and teachers attended an information session, received manuals and flashcards, and were trained to support the activities. The intervention was delivered by Kinderkinetics students under the supervision of qualified Kinderkineticists.	The intervention improved several fundamental movement skills. Statistically significant improvements were observed for galloping, sliding, two-hand striking and overhand throwing. Practical improvements were also observed in running, hopping, skipping, jumping, dribbling and underhand throwing. A 6-week intervention can improve fundamental movement skills in children aged 6–8 years and recommended further research to determine the optimal intervention duration and long-term effects.

				throwing, catching, striking, dribbling and kicking. Participants received an activity toolkit containing cones, balls, chalk, balloons, a rope, a pool noodle and beanbags.		have introduced variability in scoring.		
