# Supplementary material for: Implementation Strategies for Promoting Physical Activity in Under-Resourced South African Schools: A Scoping Review
Source: Children (Basel). 2026 Sep 22;13(10):1289. doi: 10.3390/children13101289 (PMC13703301; doi:10.3390/children13101289)
Supplement: Supplementary file 1 [file children-13-01289-s001.zip › Supplementary Table S2A. JBI Critical Appraisal of Randomized Control.pdf]

Author, Year	Q1	Q2	Q3	Q4	Q5	Q6	Q7	Q8	Q9	Q10	Q11	Q12	Q13	Overall Methodological Assessment
Nqweniso et al., 2025	Yes	Yes	No	Yes	Yes	Yes	Yes	Yes	Yes	Yes	Yes	Yes	Yes	Randomization, allocation concealment, blinding, and appropriate statistical analyses were clearly reported. Baseline differences between groups and participant attrition were considered during the analyses.
Long et al., 2022	Yes	Yes	No	Yes	Yes	Yes	Yes	Yes	Yes	Yes	Uncle ar	Yes	Yes	Cluster randomization and allocation procedures were clearly described, with blinding and standardized outcome measurements applied throughout the study. Baseline imbalances between intervention groups were addressed using adjusted statistical analyses, although intention-to-treat analysis was not explicitly reported.
Long et al., 2024	Yes	Yes	No	Yes	Yes	Yes	Yes	Yes	Yes	Yes	Uncl ear	Yes	Yes	Blinding procedures and allocation methods were clearly described, and validated measurement protocols were applied consistently across intervention groups. Loss to follow-up and baseline differences were accounted for in the analyses, although intention-to-treat analysis was not explicitly stated.
Arnaiz et al., 2023	Yes	Yes	No	No	No	Yes	Uncl ear	Yes	Yes	Yes	Yes	Yes	Yes	School-level randomization and allocation concealment were clearly described, with appropriate statistical methods used to account for clustering and repeated measurements. Baseline differences between groups were adjusted during the analyses, although blinding of participants, intervention providers, and outcome assessors was not reported.
Beckmann et al., 2022	Yes	Yes	No	Yes	Yes	Yes	Uncl ear	Yes	Yes	Yes	Yes	Yes	Yes	Outcome measurements were collected using consistent procedures across study groups. Baseline imbalances were addressed through appropriate statistical analyses, although outcome assessor blinding was not clearly described.
Nqweniso et al., 2020	Yes	Yes	Yes	No	No	Yes	Uncle ar	Yes	Yes	Yes	Yes	Yes	Yes	Outcome measurements were obtained using the same procedures across all groups, and participant attrition was reported during follow-up. Blinding of participants, intervention providers, and outcome assessors was not clearly described.
Idamokoro et al., 2024 (BMC Pediatrics)	Yes	Uncle ar	No	No	No	Yes	Uncle ar	Yes	Yes	Yes	Uncle ar	Yes	Yes	A randomized design was used, with consistent outcome assessment procedures applied across

														both groups. Baseline differences and limited reporting of allocation concealment, blinding, and intention-to-treat analysis may have introduced potential bias.
Nqweniso et al., 2021	Yes	Yes	Yes	No	No	Yes	Unclear	Yes	Yes	Yes	Unclear	Yes	Yes	Measurements were obtained using the same assessment methods across study groups. Limited reporting of blinding procedures and unclear intention-to-treat analysis may have introduced potential bias.
Van der Merwe et al., 2023	Yes	Unclear	Yes	No	No	Yes	Unclear	Yes	Yes	Yes	No	Yes	Yes	Validated assessment tools were applied consistently following random allocation. Allocation concealment and blinding were not clearly reported, and analyses were limited to participants with complete follow-up data.
Gall et al., 2020	Yes	Yes	No	No	No	Yes	Unclear	Yes	Yes	Yes	No	Yes	Yes	Data collection followed the same procedures across all study groups. Baseline differences between groups, incomplete inclusion of randomized participants in the analyses, and limited blinding may have introduced potential bias.
Gall et al., 2018	Yes	Yes	No	No	No	Yes	Unclear	Yes	Yes	Yes	No	Yes	Yes	Schools were randomly allocated to the intervention and control groups, and the same outcome measures were used throughout the study. Baseline differences between groups, limited blinding, and analyses based on participants with complete data may have introduced potential bias.
Müller et al., 2019	Yes	Yes	Yes	No	No	Yes	Unclear	Yes	Yes	Yes	No	Yes	Yes	Uniform assessment procedures were applied throughout the study. Blinding of participants and intervention providers was not feasible, and participants with incomplete follow-up data were excluded from the final analyses.
