# Supplementary material for: Implementation Strategies for Promoting Physical Activity in Under-Resourced South African Schools: A Scoping Review
Source: Children (Basel). 2026 Sep 22;13(10):1289. doi: 10.3390/children13101289 (PMC13703301; doi:10.3390/children13101289)
Supplement: Supplementary file 1 [file children-13-01289-s001.zip › Supplementary Table S2B. JBI Critical Appraisal of Quasi-Experimental.pdf]

Author, Year	Q1	Q2	Q3	Q4	Q5	Q6	Q7	Q8	Q9	Overall Methodological Assessment
Zimu et al., 2024	Yes	Yes	No	Yes	Yes	Yes	Yes	Yes	Yes	Participants were assessed before and after the programme using identical outcome measures. The non-randomized design and differences between the treatment and control groups may have influenced the observed outcomes.
Tian, du Toit & Toriola, 2017	Yes	Yes	No	Yes	Yes	Yes	Yes	Yes	Yes	Physical activity was assessed before and after the intervention using the same measurement approach. Convenience sampling and the lack of random allocation of schools may have affected the comparability of the intervention and control groups.
Tian, du Toit & Toriola, 2014	Yes	Yes	No	Yes	Yes	Yes	Yes	Yes	Yes	Physical and motor fitness outcomes were measured before and after the intervention using identical assessment methods. Convenience sampling and the allocation of schools without randomization may have affected the comparability of the study groups.
Idamokoro et al., 2024 (Quasi) SAJCE	Yes	Yes	No	Yes	Yes	Yes	Yes	Yes	Yes	Fundamental movement skills were assessed before, immediately after, and six months following the intervention using standardized procedures. The non-random allocation of schools and differences in care between the comparison groups may have influenced the observed outcomes.
Africa & Van Deventer, 2015	Yes	Yes	Yes	Yes	Yes	Yes	Yes	Yes	Yes	Balance and bilateral coordination were assessed before and after the 12-week intervention using standardized measurement procedures. The quasi-experimental design and convenience selection of the study school may have introduced selection bias.
Idamokoro et al., 2023	Yes	Yes	No	Yes	Yes	Yes	Yes	Yes	Yes	Body composition outcomes were measured before, immediately after, and six months following the intervention using standardized assessment procedures. The non-randomized design and differences in care between the comparison groups may have influenced the observed outcomes.
Ferguson et al., 2015	Yes	Yes	Yes	Yes	Yes	Yes	Yes	Yes	Yes	Motor performance and physical fitness were assessed before and after the 9-week intervention using standardized assessment procedures. The quasi-experimental design and

										purposive sampling may have introduced selection bias.
Africa, van Stryp & Vermaak, 2026	Yes	Yes	No	Yes	Yes	Yes	Yes	Yes	Yes	Fundamental motor skills were evaluated at baseline and following the 6-week intervention using standardized assessment procedures. The quasi-experimental design and differences in care between the comparison groups may have influenced the observed outcomes.
Fisher & Africa, 2023	Yes	Yes	No	Yes	Yes	Yes	Yes	Yes	Yes	Physical fitness and mathematics performance were assessed at baseline and after the 16-week intervention using standardized assessment procedures. The quasi-experimental design and differences in care between the comparison groups may have influenced the observed outcomes.
Williams, 2018	Yes	N/A	N/A	No	Yes	Yes	Yes	Yes	Yes	Study outcomes were assessed before and after the health promotion intervention using standardized assessment procedures. The pretest–posttest design without a comparison group limited attribution of the observed changes to the intervention alone.
Pienaar et al., 2012	Yes	Yes	No	Yes	Yes	Yes	Yes	No	Yes	Participants were assessed at baseline and at multiple subsequent testing opportunities using the same physical activity outcome measure. The quasi-experimental design, baseline differences between groups, and incomplete measurement of the control group across all testing opportunities may have influenced the observed outcomes.
