# Supplementary material for: Implementation Strategies for Promoting Physical Activity in Under-Resourced South African Schools: A Scoping Review
Source: Children (Basel). 2026 Sep 22;13(10):1289. doi: 10.3390/children13101289 (PMC13703301; doi:10.3390/children13101289)
Supplement: Supplementary file 1 [file children-13-01289-s001.zip › Supplementary Table S2C. JBI Critical Appraisal of Cohort Studies.pdf]

Author, Year	Q1	Q2	Q3	Q4	Q5	Q6	Q7	Q8	Q9	Q10	Q11	Overall Methodological Assessment
Fuller et al., 2010	Yes	Yes	Yes	Yes	Yes	Yes	Yes	Yes	Yes	No	Yes	Health knowledge outcomes were measured at baseline, post-intervention, and follow-up using standardized assessment procedures. Comparable study groups and appropriate statistical analyses were reported, although strategies to address incomplete follow-up were not described.
