# Supplementary material for: Implementation Strategies for Promoting Physical Activity in Under-Resourced South African Schools: A Scoping Review
Source: Children (Basel). 2026 Sep 22;13(10):1289. doi: 10.3390/children13101289 (PMC13703301; doi:10.3390/children13101289)
Supplement: Supplementary file 1 [file children-13-01289-s001.zip › Supplementary Table S3. full search strategies for all databases.pdf]

Databases	Search string	Filter	Results
**PubMed**	(<br>"Implementation Science"[MeSH Terms]<br>OR implementation [Title/Abstract]<br>OR intervention*[Title/Abstract]<br>OR strateg*[Title/Abstract]<br>OR promotion [Title/Abstract]<br>OR "health promotion"[Title/Abstract]<br>OR program*[Title/Abstract]<br>OR programme*[Title/Abstract]<br>OR initiative*[Title/Abstract]<br>OR "implementation science"[Title/Abstract]<br>)<br><br>AND<br><br>(<br>"Motor Activity"[MeSH Terms]<br>OR "Exercise"[MeSH Terms]<br>OR "Sports"[MeSH Terms]<br>OR "Physical Education and Training"[MeSH Terms]<br>OR "physical activity"[Title/Abstract]<br>OR exercise [Title/Abstract]<br>OR sport*[Title/Abstract]<br>OR "sport participation"[Title/Abstract]<br>OR "physical education"[Title/Abstract]<br>OR PE[Title/Abstract]<br>OR "physical literacy"[Title/Abstract]<br>OR "motor competence"[Title/Abstract]<br>OR "motor skill*"[Title/Abstract]<br>OR "movement skill*"[Title/Abstract]<br>OR "fundamental movement skill*"[Title/Abstract]<br>OR FMS[Title/Abstract]<br>OR "active classroom*"[Title/Abstract]	2010-2026<br>English	2826

	OR "active school*"[Title/Abstract]<br>OR "Life Orientation"[Title/Abstract]<br>OR LO[Title/Abstract]<br>OR "school health"[Title/Abstract]<br>OR "school wellness"[Title/Abstract]<br>)<br><br>AND<br><br>(<br>"Child"[MeSH Terms]<br>OR "Adolescent"[MeSH Terms]<br>OR "Students"[MeSH Terms]<br>OR learner*[Title/Abstract]<br>OR child*[Title/Abstract]<br>OR children [Title/Abstract]<br>OR student*[Title/Abstract]<br>OR adolescent*[Title/Abstract]<br>OR youth[Title/Abstract]<br>)<br><br>AND<br><br>(<br>"Socioeconomic Factors"[MeSH Terms]<br>OR "Poverty Areas"[MeSH Terms]<br>OR "Rural Population"[MeSH Terms]<br>OR "under-resourced"[Title/Abstract]<br>OR "low-income"[Title/Abstract]<br>OR "low-resource"[Title/Abstract]<br>OR rural [Title/Abstract]<br>OR disadvantaged [Title/Abstract]<br>OR underserved [Title/Abstract]<br>OR township*[Title/Abstract]		

	OR "resource-constrained"[Title/Abstract]<br><br>OR "resource poor"[Title/Abstract]<br><br>OR "low socioeconomic"[Title/Abstract]<br><br>OR "low SES"[Title/Abstract]<br><br>OR poverty [Title/Abstract]<br><br>OR impoverished [Title/Abstract]<br><br>OR quintile*[Title/Abstract]<br><br>OR "no-fee school*"[Title/Abstract]<br><br>)<br><br><br>AND<br><br><br>(<br><br>"Schools"[MeSH Terms]<br><br>OR school*[Title/Abstract]		
Web of Science	TS=<br><br>(<br><br>(implementation OR intervention* OR strateg* OR promotion OR "health promotion" OR program* OR programme* OR initiative* OR "implementation science")<br><br>AND<br><br>("physical activity" OR exercise OR sport* OR "sport participation" OR "physical education" OR PE OR "physical literacy" OR "motor competence" OR "motor skill*" OR "movement skill*" OR "fundamental movement skill*" OR FMS OR "active classroom*" OR "active school*" OR "Life Orientation" OR LO OR "school health" OR "school wellness")<br><br>AND<br><br>(learner* OR child* OR children OR student* OR adolescent* OR youth)<br><br>AND<br><br>("under-resourced" OR "low-income" OR "low-resource" OR rural OR disadvantaged OR underserved OR township* OR "resource-constrained" OR "resource poor" OR "low socioeconomic" OR "low SES" OR poverty OR impoverished OR quintile* OR "no-fee school*")<br><br>AND<br><br>(school*)	2010-2026<br><br>English	278

Scopus	TITLE-ABS-KEY<br>(<br>(implementation OR intervention* OR strateg* OR promotion OR "health promotion" OR program* OR programme* OR initiative* OR "implementation science")<br>AND<br>("physical activity" OR exercise OR sport* OR "sport participation" OR "physical education" OR PE OR "physical literacy" OR "motor competence" OR "motor skill*" OR "movement skill*" OR "fundamental movement skill*" OR FMS OR "active classroom*" OR "active school*" OR "Life Orientation" OR LO OR "school health" OR "school wellness")<br>AND<br>(learner* OR child* OR children OR student* OR adolescent* OR youth)<br>AND<br>("under-resourced" OR "low-income" OR "low-resource" OR rural OR disadvantaged OR underserved OR township* OR "resource-constrained" OR "resource poor" OR "low socioeconomic" OR "low SES" OR poverty OR impoverished OR quintile* OR "no-fee school*")<br>AND<br>(school*)	2010-2026<br>English	7942
EBSCOHost	TX<br>(<br>(implementation OR intervention* OR strateg* OR promotion OR "health promotion" OR program* OR programme* OR initiative* OR "implementation science")<br>AND<br>("physical activity" OR exercise OR sport* OR "sport participation" OR "physical education" OR PE OR "physical literacy" OR "motor competence" OR "motor skill*" OR "movement skill*" OR "fundamental movement skill*" OR FMS OR "active classroom*" OR "active school*" OR "Life Orientation" OR LO OR "school health" OR "school wellness")<br>AND<br>(learner* OR child* OR children OR student* OR adolescent* OR youth)<br>AND<br>("under-resourced" OR "low-income" OR "low-resource" OR rural OR disadvantaged OR underserved OR township* OR "resource-constrained" OR "resource poor" OR "low socioeconomic" OR "low SES" OR poverty OR impoverished OR quintile* OR "no-fee school*")<br>AND<br>(school*)	2010-2026<br>English<br>Academic Search complete; ERIC; SPORTDiscus ; CINAHL	4199
Google Scholar	Search No.1<br>Title words: physical activity	2010-2026<br>English	1213

	Keywords: intervention* implementation strateg* program* programme* initiative* trial school South Africa<br>Search No.2<br>physical education<br>intervention* implementation strateg* program* programme* initiative* trial school South Africa<br>Search No.3<br>Title words: physical literacy<br>Keywords: intervention* implementation strateg* program* programme* initiative* trial school South Africa<br>Search No.4<br>Title words: fundamental movement skills<br>Keywords: intervention* implementation strateg* program* programme* initiative* trial school South Africa<br>Search No.5<br>Title words: Sport participation<br>Keywords: intervention* implementation strateg* program* programme* initiative* trial school South Africa	The search was conducted using the Publish or Perish software	
