# Supplementary material for: Implementation Strategies for Promoting Physical Activity in Under-Resourced South African Schools: A Scoping Review
Source: Children (Basel). 2026 Sep 22;13(10):1289. doi: 10.3390/children13101289 (PMC13703301; doi:10.3390/children13101289)
Supplement: Supplementary file 1 [file children-13-01289-s001.zip › Supplementary Table S4. Programme Clusters and Sample Overlap.pdf]

Supplementary Table S4. Programme Clusters and Sample Overlap

Programme	Linked publications	Province	Same schools/sample?	Unique school sites/overlap status
**KaziKidz/KaziAfya**	Nqweniso et al., 2025; Long et al., 2022; Long et al., 2024; Beckmann et al., 2022; Van der Merwe et al., 2023	Eastern Cape; Free State	Programme overlap identified. Long et al. (2022) and Long et al. (2024) report the same sample characteristics (1,304 children, Grades 1–4, Gqeberha). The other publications describe KaziKidz/KaziAfya interventions, but the available information does not establish that they used the same schools or participants.	Not determinable from the reported study information. Confirmed overlap exists between Long et al. (2022) and Long et al. (2024), but the number of unique school sites across all linked publications cannot be established. Van der Merwe et al. (2023) used the KaziKidz toolkit but was not conducted as part of the KaziAfya trial in Gqeberha.
**Multicomponent school-based physical activity programme**	Gall et al., 2018; Gall et al., 2020; Müller et al., 2019; Nqweniso et al., 2020; Nqweniso et al., 2021 ; Arnaiz et al., 2023	Eastern Cape	Same schools identified; same sample not established. The publications describe eight disadvantaged/Quintile 3 primary schools in Port Elizabeth/Gqeberha and highly similar school-based PA components. The available information does not confirm that all publications used the same participants.	Eight school sites are identified across the linked publications; participant overlap cannot be confirmed from the reported study information.
**Fundamental Movement Skills (FMS)/SPARK-based programme**	Idamokoro et al., 2023; Idamokoro et al., 2024 SAJCE<br>Idamokoro et al., 2024 (BMC Pediatrics	Eastern Cape	Idamokoro et al. (2023) and Idamokoro et al. (2024) (SAJCE) report outcomes from the same cohort of Grade 2 learners from three rural primary schools in Raymond Mhlaba Municipality (N = 93; intervention = 57, control = 36) who participated in the same 9-week FMS/SPARK-based intervention programme.<br>In contrast, Idamokoro et al. (2024) (BMC Pediatrics) reported physical activity outcomes from a separate subsample of participants with complete accelerometer data (N = 70; intervention = 35, control = 35) derived from the same broader intervention project	Three rural primary schools in Raymond Mhlaba Municipality. The 2023 and 2024 (SAJCE) publications reported the same participant cohort, while the 2024 (*BMC Pediatrics*) publication reported a different participant sample from the same intervention programme.
**Enhanced Quality Physical Education Programme**	Tian, du Toit & Toriola, 2014; Tian, du Toit & Toriola, 2017	Northwest	Same schools/sample. Both publications report on 110 Grade 7 learners from two primary schools in Potchefstroom (experimental = 40; control = 70) and the same 12-week enhanced-quality Physical Education programme.	Two school sites; same participant sample reported.

**Other distinct programmes**	Fuller et al., 2010; Zimu et al., 2024; Pienaar et al., 2012; Williams, 2018; Africa & Van Deventer, 2015; Ferguson et al., 2015; Fisher & Africa, 2023; Africa, van Stryp & Vermaak, 2026	Western Cape; KwaZulu-Natal; Northwest; Gauteng	No overlap was identified from the available reports. These publications describe distinct programmes, settings and/or populations and do not provide evidence of shared schools or samples.	Not determinable from the reported study information. No shared school sites or participant samples were identified, but the available reports do not permit a definitive count of unique school sites across these publications.

Because school identifiers were not consistently reported across publications, a definitive count of unique school sites could not be established for all programme clusters. Where shared schools or participant samples could be confirmed from the available reports, this is indicated; otherwise, overlap is reported as uncertain rather than assumed.
